# Supplementary figures and images for: Intraoperative 3D quantitative magnetic resonance imaging in paediatric brain tumour surgery
Source: PLoS One. 2026 Feb 17;21(2):e0332562. doi: 10.1371/journal.pone.0332562 (PMC12912603; doi:10.1371/journal.pone.0332562)

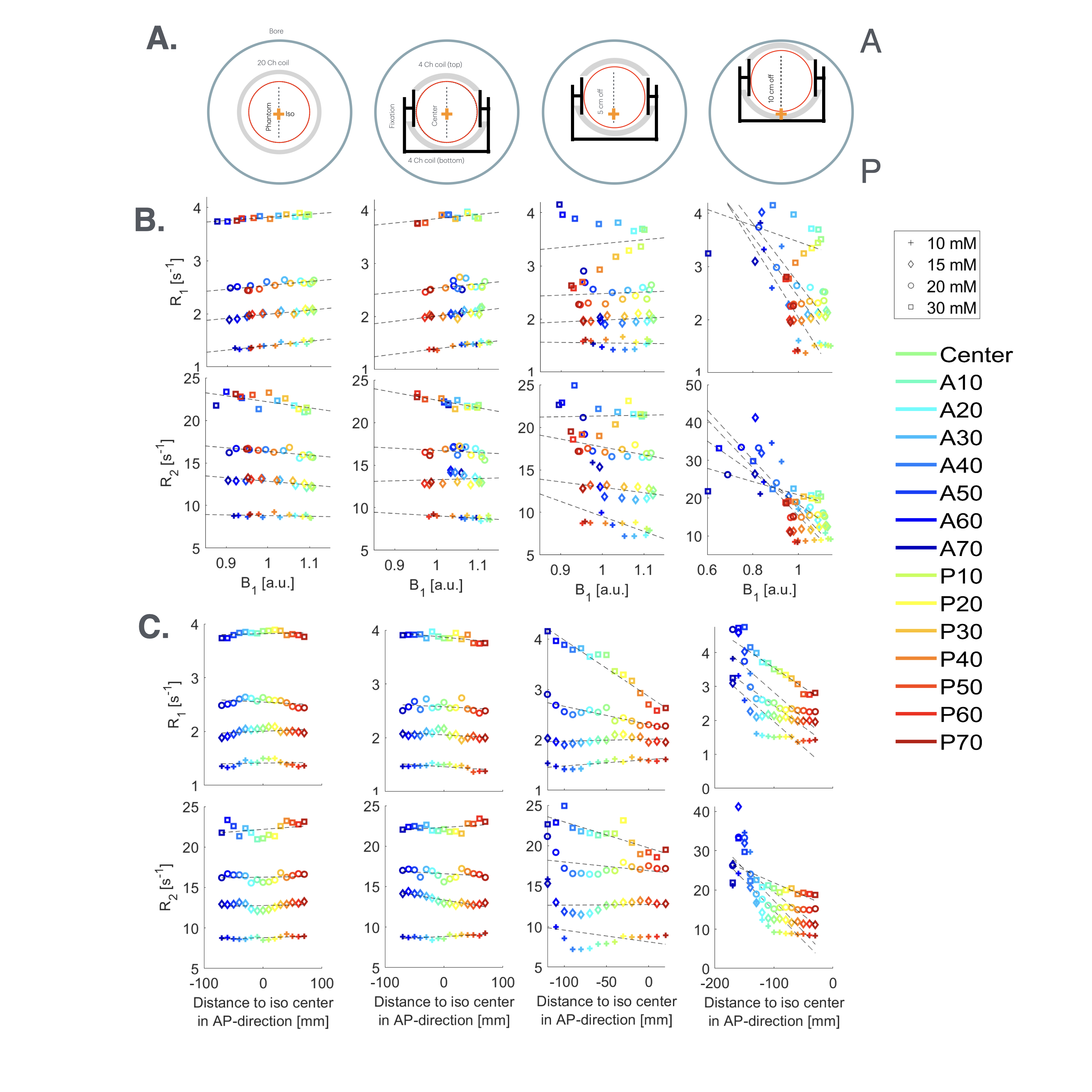

Supplement: S1 Fig — (A) The measurements were performed in four different configurations. The left panel shows one of the four phantoms (10, 25, 20 and 30 mM) centred within a head/neck coil (coil in grey, and phantom in red). The three setups to the right are with a phantom and the two flex coils positioned at 0 cm from the centre, at 5 cm from the centre and 10 cm from the centre, respectively. The phantom was placed in the head fixation. (B) R1 and R2 (s-1) respectively, as a function of B1+ (in a.u., where 1.0 represents an expected B1+ vs flip angle relation). Four different phantoms were used, placed in different positions with respect to the isocentre. The ROI positions within each phantom varied from A70 to P70. B1+ were typically in the range 0.9 to 1.1, except in the more extreme phantom positions. (C) R1 and R2 (s-1) respectively, as a function of bore position of the ROIs. The ROI positions within each phantom varied from A70 to P70. N.B. The cases when the phantoms and ROIs were placed extremely far off centre (although within the max FOV of the scanner) resulted in large offsets. (TIFF) [file pone.0332562.s001.tiff]

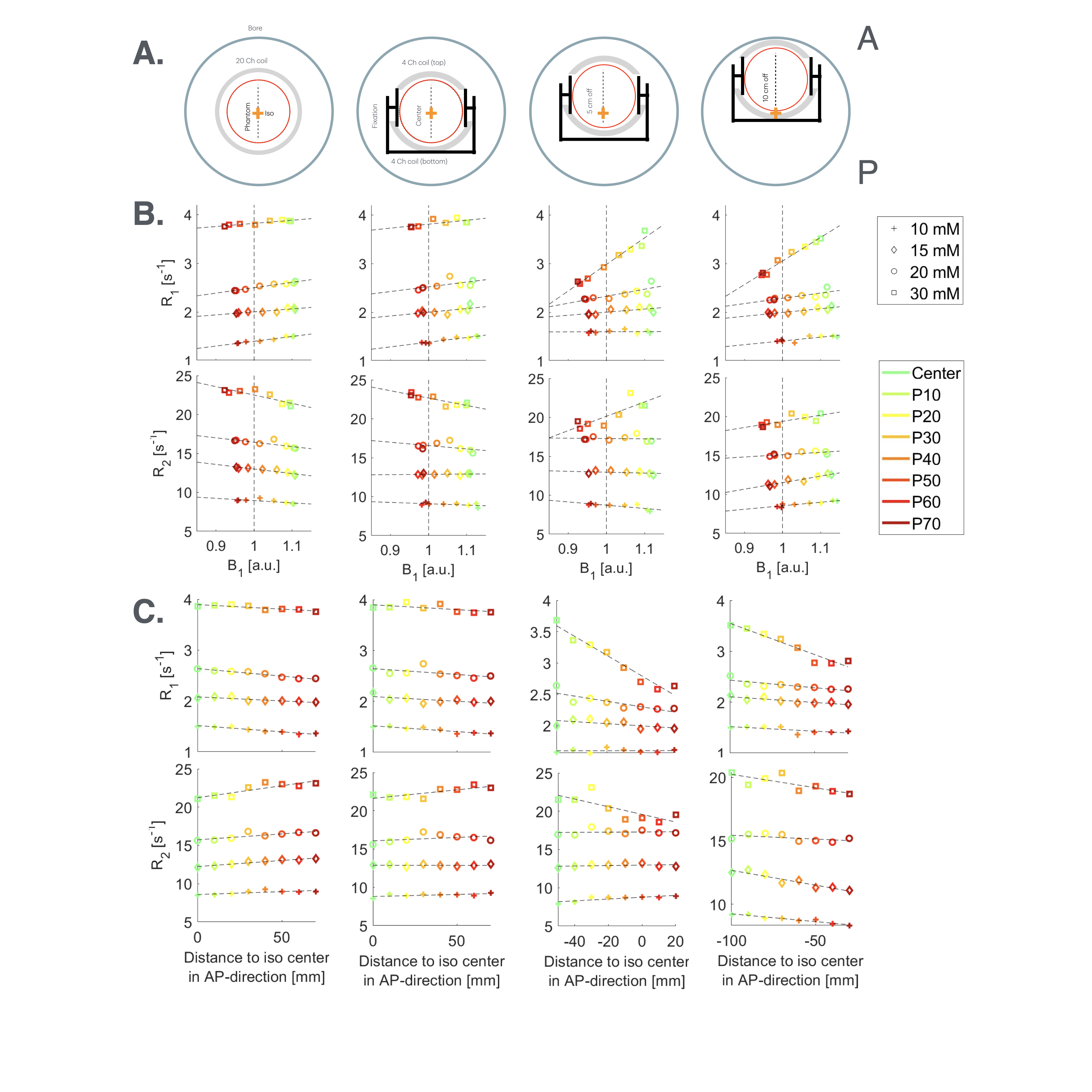

Supplement: S2 Fig — (A) The measurements were performed in four different configurations. The left panel shows one of the four phantoms (10, 25, 20 and 30 mM) centred within a head/neck coil (coil in grey, and phantom in red). The three setups to the right are with a phantom and the two flex coils positioned at 0 cm from the centre, at 5 cm from the centre and 10 cm from the centre, respectively. The phantom was placed in the head fixation. (B) R1 and R2 (s-1) respectively, as a function of B1+ (in a.u., where 1.0 represents an expected B1+ vs flip angle relation). Four different phantoms were used, placed in different positions with respect to the isocentre. The ROI positions within each phantom varied from centre to P70. B1+ were typically in the range 0.9 to 1.1. (C) R1 and R2 (s-1) respectively, as a function of bore position of the ROIs. The ROI positions within each phantom varied from centre to P70. N.B. Excluding anterior ROIs only small variation are observed. (TIFF) [file pone.0332562.s002.tiff]

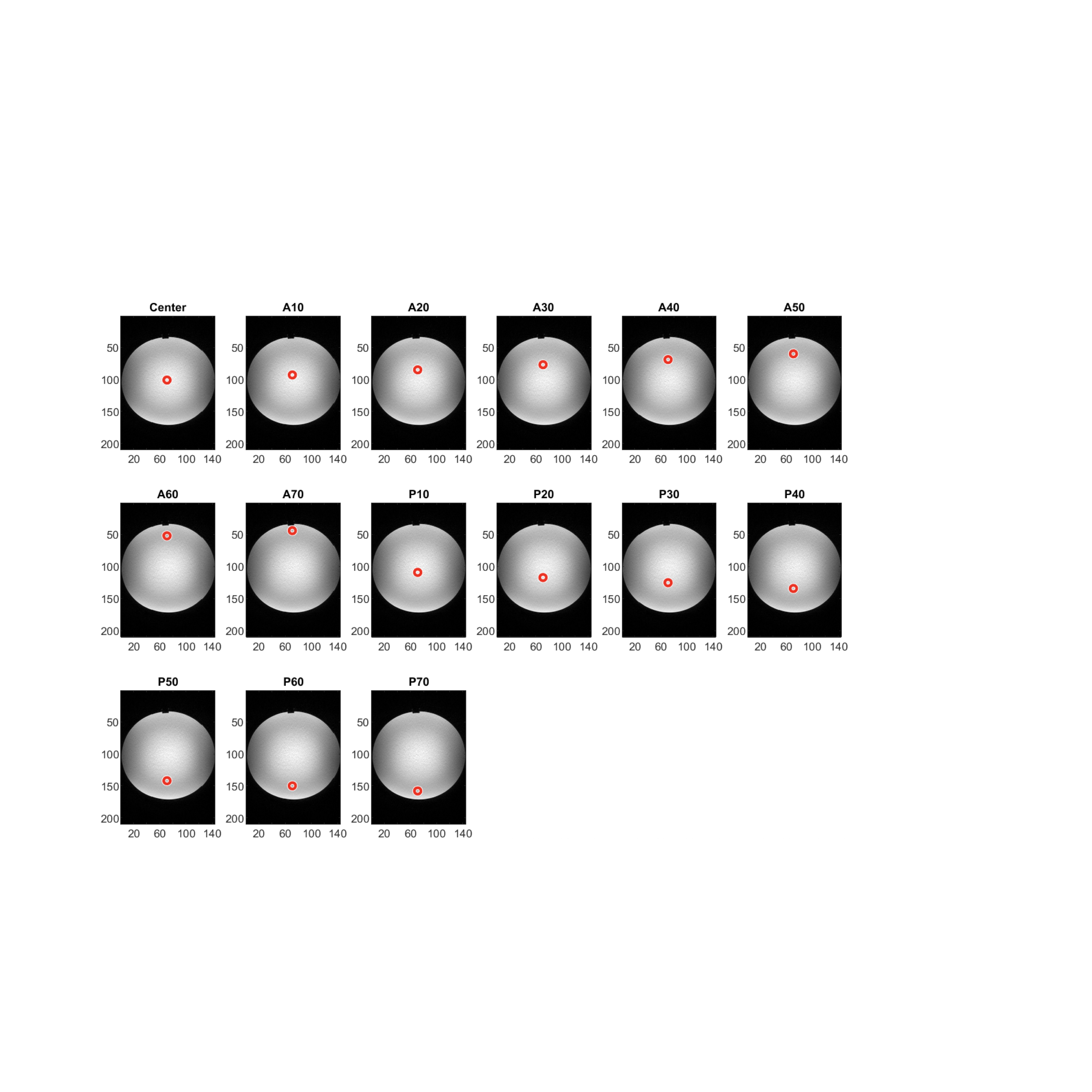

Supplement: S3 Fig — All ROI placements in the AP direction in the phantoms. The images are shown in axial view. (TIFF) [file pone.0332562.s003.tiff]
