# Supplementary material for: Intraoperative 3D quantitative magnetic resonance imaging in paediatric brain tumour surgery
Source: PLoS One. 2026 Feb 17;21(2):e0332562. doi: 10.1371/journal.pone.0332562 (PMC12912603; doi:10.1371/journal.pone.0332562)
Supplement: S1 Data — (PDF) [file pone.0332562.s004.pdf]

Preoperative QALAS protocol 1.2x1.2x1.2 mm³

TA: 6:06 PM: FIX Voxel size: 1.2×1.2×1.2 mmPAT: 3 Rel. SNR: 1.00 : qalas

Properties

Prio recon	Off
Load images to viewer	On
Inline movie	Off
Auto store images	On
Load images to stamp segments	Off
Load images to graphic segments	Off
Auto open inline display	Off
Auto close inline display	Off
Start measurement without further preparation	Off
Wait for user to start	Off
Start measurements	Single measurement

Routine

Slab group	1
Slabs	1
Dist. factor	50 %
Position	L3.2 A2.6 H0.3 mm
Orientation	Sagittal
Phase enc. dir.	A >> P
AutoAlign	Head > Basis
Phase oversampling	0 %
Slice oversampling	0.0 %
Slices per slab	144
FoV read	256 mm
FoV phase	101.0 %
Slice thickness	1.20 mm
TR	908.40 ms
TE	2.33 ms
Averages	1
Concatenations	1
Filter	Distortion Corr.(3D), Prescan Normalize
Coil elements	HE1-4;NE1,2

Contrast - Common

TR	908.40 ms
TE	2.33 ms
Flip angle	4 deg
Fat suppr.	None
Water suppr.	None

Contrast - Dynamic

Averages	1
Averaging mode	Long term
Reconstruction	Magn./Phase
Multiple series	Off

Resolution - Common

FoV read	256 mm
FoV phase	101.0 %
Slice thickness	1.20 mm
Base resolution	208
Phase resolution	100 %
Slice resolution	100 %
Phase partial Fourier	Off
Slice partial Fourier	Off
Interpolation	Off

Resolution - iPAT

PAT mode	GRAPPA
Accel. factor PE	3
Ref. lines PE	36
Accel. factor 3D	1
Reference scan mode	Integrated

Resolution - Filter Image

Image Filter	Off
Distortion Corr.	On
Mode	3D
Unfiltered images	On
Prescan Normalize	On
Unfiltered images	Off
Normalize	Off
B1 filter	Off

Resolution - Filter Rawdata

Raw filter	Off
Elliptical filter	Off

Geometry - Common

Slab group	1
Slabs	1
Dist. factor	50 %
Position	L3.2 A2.6 H0.3 mm
Orientation	Sagittal
Phase enc. dir.	A >> P
Slice oversampling	0.0 %
Slices per slab	144
FoV read	256 mm
FoV phase	101.0 %
Slice thickness	1.20 mm
TR	908.40 ms
Multi-slice mode	Sequential
Series	Interleaved
Concatenations	1

Geometry - AutoAlign

Slab group	1
Position	L3.2 A2.6 H0.3 mm
Orientation	Sagittal
Phase enc. dir.	A >> P
AutoAlign	Head > Basis
Initial Position	L3.2 A2.6 H0.3
L	3.2 mm
A	2.6 mm
H	0.3 mm
Initial Rotation	0.00 deg
Initial Orientation	Sagittal

Geometry - Navigator

System - Miscellaneous

Positioning mode	FIX
Table position	H
Table position	0 mm
MSMA	S - C - T
Sagittal	R >> L
Coronal	A >> P
Transversal	F >> H

System - Miscellaneous

Coil Combine Mode	Adaptive Combine
Save uncombined	Off
Matrix Optimization	Off
AutoAlign	Head > Basis
Coil Select Mode	On - AutoCoilSelect

System - Adjustments

B0 Shim mode	Brain
B1 Shim mode	TrueForm
Adjust with body coil	Off
Confirm freq. adjustment	Off
Assume Dominant Fat	Off
Assume Silicone	Off
Adjustment Tolerance	Auto

System - Adjust Volume

Position	L3.2 A2.6 H0.3 mm
Orientation	Sagittal
Rotation	90.00 deg
F >> H	256 mm
A >> P	259 mm
R >> L	173 mm
Reset	Off

System - pTx Volumes

B1 Shim mode	TrueForm
Excitation	Slab-sel.

System - Tx/Rx

Frequency 1H	123.254412 MHz
Correction factor	1
Gain	Low
Img. Scale Cor.	1.000
Reset	Off
? Ref. amplitude 1H	0.000 V

Physio - Signal1

1st Signal/Mode	None
TR	908.40 ms
Concatenations	1

Physio - Cardiac

Fat suppr.	None
Dark blood	Off
FoV read	256 mm
FoV phase	101.0 %
Phase resolution	100 %

Physio - PACE

Resp. control	Off
Concatenations	1

Inline - Common

Subtract	Off
StdDev	Off
Save original images	On

Inline - MIP

MIP-Sag	Off
MIP-Cor	Off
MIP-Tra	Off
MIP-Time	Off
Save original images	On

Inline - Composing

Distortion Corr.	On
Mode	3D
Unfiltered images	On

Inline - MapIt

Save original images	On
MapIt	None
Flip angle	4 deg
TR	908.40 ms
TE	2.33 ms

Sequence - Part 1

Introduction	Off
Dimension	3D
Elliptical scanning	On
Reordering	Radial
Asymmetric echo	Off
Flow comp.	No
Multi-slice mode	Sequential
Echo spacing	5.6 ms
Bandwidth	440 Hz/Px

Sequence - Part 2

RF pulse type	Fast
Gradient mode	Fast
Excitation	Slab-sel.
RF spoiling	On
Incr. Gradient spoiling	On
Turbo factor	140

Sequence - Assistant

Mode	Off

Preoperative QALAS protocol 1.2x1.2x1.2 mm3 with GD

TA: 6:06 PM: FIX Voxel size: 1.2×1.2×1.2 mmPAT: 3 Rel. SNR: 1.00 : qalas

Properties

Prio recon	Off
Load images to viewer	On
Inline movie	Off
Auto store images	On
Load images to stamp segments	Off
Load images to graphic segments	Off
Auto open inline display	Off
Auto close inline display	Off
Start measurement without further preparation	Off
Wait for user to start	Off
Start measurements	Single measurement

Routine

Slab group	1
Slabs	1
Dist. factor	50 %
Position	L3.2 A2.6 H0.3 mm
Orientation	Sagittal
Phase enc. dir.	A >> P
AutoAlign	Head > Basis
Phase oversampling	0 %
Slice oversampling	0.0 %
Slices per slab	144
FoV read	256 mm
FoV phase	101.0 %
Slice thickness	1.20 mm
TR	908.40 ms
TE	2.33 ms
Averages	1
Concatenations	1
Filter	Distortion Corr.(3D), Prescan Normalize
Coil elements	HE1-4;NE1,2

Contrast - Common

TR	908.40 ms
TE	2.33 ms
Flip angle	4 deg
Fat suppr.	None
Water suppr.	None

Contrast - Dynamic

Averages	1
Averaging mode	Long term
Reconstruction	Magn./Phase
Multiple series	Off

Resolution - Common

FoV read	256 mm
FoV phase	101.0 %
Slice thickness	1.20 mm
Base resolution	208
Phase resolution	100 %
Slice resolution	100 %
Phase partial Fourier	Off
Slice partial Fourier	Off
Interpolation	Off

Resolution - iPAT

PAT mode	GRAPPA
Accel. factor PE	3
Ref. lines PE	36
Accel. factor 3D	1
Reference scan mode	Integrated

Resolution - Filter Image

Image Filter	Off
Distortion Corr.	On
Mode	3D
Unfiltered images	On
Prescan Normalize	On
Unfiltered images	Off
Normalize	Off
B1 filter	Off

Resolution - Filter Rawdata

Raw filter	Off
Elliptical filter	Off

Geometry - Common

Slab group	1
Slabs	1
Dist. factor	50 %
Position	L3.2 A2.6 H0.3 mm
Orientation	Sagittal
Phase enc. dir.	A >> P
Slice oversampling	0.0 %
Slices per slab	144
FoV read	256 mm
FoV phase	101.0 %
Slice thickness	1.20 mm
TR	908.40 ms
Multi-slice mode	Sequential
Series	Interleaved
Concatenations	1

Geometry - AutoAlign

Slab group	1
Position	L3.2 A2.6 H0.3 mm
Orientation	Sagittal
Phase enc. dir.	A >> P
AutoAlign	Head > Basis
Initial Position	L3.2 A2.6 H0.3
L	3.2 mm
A	2.6 mm
H	0.3 mm
Initial Rotation	0.00 deg
Initial Orientation	Sagittal

Geometry - Navigator

System - Miscellaneous

Positioning mode	FIX
Table position	H
Table position	0 mm
MSMA	S - C - T
Sagittal	R >> L
Coronal	A >> P
Transversal	F >> H

System - Miscellaneous

Coil Combine Mode	Adaptive Combine
Save uncombined	Off
Matrix Optimization	Off
AutoAlign	Head > Basis
Coil Select Mode	On - AutoCoilSelect

System - Adjustments

B0 Shim mode	Brain
B1 Shim mode	TrueForm
Adjust with body coil	Off
Confirm freq. adjustment	Off
Assume Dominant Fat	Off
Assume Silicone	Off
Adjustment Tolerance	Auto

System - Adjust Volume

Position	L3.2 A2.6 H0.3 mm
Orientation	Sagittal
Rotation	90.00 deg
F >> H	256 mm
A >> P	259 mm
R >> L	173 mm
Reset	Off

System - pTx Volumes

B1 Shim mode	TrueForm
Excitation	Slab-sel.

System - Tx/Rx

Frequency 1H	123.254412 MHz
Correction factor	1
Gain	Low
Img. Scale Cor.	1.000
Reset	Off
? Ref. amplitude 1H	0.000 V

Physio - Signal1

1st Signal/Mode	None
TR	908.40 ms
Concatenations	1

Physio - Cardiac

Fat suppr.	None
Dark blood	Off
FoV read	256 mm
FoV phase	101.0 %
Phase resolution	100 %

Physio - PACE

Resp. control	Off
Concatenations	1

Inline - Common

Subtract	Off
StdDev	Off
Save original images	On

Inline - MIP

MIP-Sag	Off
MIP-Cor	Off
MIP-Tra	Off
MIP-Time	Off
Save original images	On

Inline - Composing

Distortion Corr.	On
Mode	3D
Unfiltered images	On

Inline - MapIt

Save original images	On
MapIt	None
Flip angle	4 deg
TR	908.40 ms
TE	2.33 ms

Sequence - Part 1

Introduction	Off
Dimension	3D
Elliptical scanning	On
Reordering	Radial
Asymmetric echo	Off
Flow comp.	No
Multi-slice mode	Sequential
Echo spacing	5.6 ms
Bandwidth	440 Hz/Px

Sequence - Part 2

RF pulse type	Fast
Gradient mode	Fast
Excitation	Slab-sel.
RF spoiling	On
Incr. Gradient spoiling	On
Turbo factor	140

Sequence - Assistant

Mode	Off

Preoperative B1map
TA: 0:12 PM: FIX Voxel size: 7.0×7.0×8.0 mmPAT: Off Rel. SNR: 1.00 : tfl

Properties

Prio recon	Off
Load images to viewer	On
Inline movie	Off
Auto store images	On
Load images to stamp segments	Off
Load images to graphic segments	Off
Auto open inline display	Off
Auto close inline display	Off
Start measurement without further preparation	Off
Wait for user to start	Off
Start measurements	Single measurement

Routine

Slice group	1
Slices	18
Dist. factor	100 %
Position	L3.2 A2.6 H0.3 mm
Orientation	Sagittal
Phase enc. dir.	A >> P
AutoAlign	Head > Basis
Phase oversampling	0 %
FoV read	450 mm
FoV phase	100.0 %
Slice thickness	8.0 mm
TR	5000.0 ms
TE	1.83 ms
Averages	1
Concatenations	1
Filter	None
Coil elements	HE1-4;NE1,2

Contrast - Common

TR	5000.0 ms
TE	1.83 ms
Magn. preparation	None
Flip angle	8 deg
Fat suppr.	None
Water suppr.	None

Contrast - Dynamic

Averages	1
Reconstruction	Magnitude
Measurements	1
Multiple series	Each measurement

Resolution - Common

FoV read	450 mm
FoV phase	100.0 %
Slice thickness	8.0 mm
Base resolution	64
Phase resolution	100 %
Phase partial Fourier	Off
Interpolation	Off

Resolution - iPAT

PAT mode	None

Resolution - Filter Image

Image Filter	Off

Resolution - Filter Image

Distortion Corr.	Off
Prescan Normalize	Off
Normalize	Off
B1 filter	Off

Resolution - Filter Rawdata

Raw filter	Off
Elliptical filter	Off

Geometry - Common

Slice group	1
Slices	18
Dist. factor	100 %
Position	L3.2 A2.6 H0.3 mm
Orientation	Sagittal
Phase enc. dir.	A >> P
FoV read	450 mm
FoV phase	100.0 %
Slice thickness	8.0 mm
TR	5000.0 ms
Multi-slice mode	Interleaved
Series	Interleaved
Concatenations	1

Geometry - AutoAlign

Slice group	1
Position	L3.2 A2.6 H0.3 mm
Orientation	Sagittal
Phase enc. dir.	A >> P
AutoAlign	Head > Basis
Initial Position	L3.2 A2.6 H0.3
L	3.2 mm
A	2.6 mm
H	0.3 mm
Initial Rotation	0.00 deg
Initial Orientation	Sagittal

System - Miscellaneous

Positioning mode	FIX
Table position	H
Table position	0 mm
MSMA	S - C - T
Sagittal	R >> L
Coronal	A >> P
Transversal	F >> H
Coil Combine Mode	Sum of Squares
Save uncombined	Off
Matrix Optimization	Off
AutoAlign	Head > Basis
Coil Select Mode	Default

System - Adjustments

B0 Shim mode	Tune up
B1 Shim mode	TrueForm
Adjust with body coil	Off
Confirm freq. adjustment	Off
Assume Dominant Fat	Off
Assume Silicone	Off
Adjustment Tolerance	Auto

System - Adjust Volume

Position	Isocenter
Orientation	Transversal
Rotation	0.00 deg
A >> P	263 mm
R >> L	350 mm
F >> H	350 mm
Reset	Off

System - pTx Volumes

B1 Shim mode	TrueForm
Excitation	Slice-sel.

System - Tx/Rx

Frequency 1H	123.254412 MHz
Correction factor	1
Gain	High
Img. Scale Cor.	1.000
Reset	Off
? Ref. amplitude 1H	0.000 V

Inline - Common

Subtract	Off
Measurements	1
StdDev	Off
Save original images	On

Inline - MIP

MIP-Sag	Off
MIP-Cor	Off
MIP-Tra	Off
MIP-Time	Off
Save original images	On

Inline - Composing

Distortion Corr.	Off

Sequence - Part 1

Introduction	On
Dimension	2D
Asymmetric echo	Allowed
Flow comp.	No
Multi-slice mode	Interleaved
Echo spacing	4.1 ms
Bandwidth	490 Hz/Px

Sequence - Part 2

RF pulse type	Low SAR
Gradient mode	Normal
Excitation	Slice-sel.
RF spoiling	On
Turbo factor	64

Sequence - Assistant

Mode	Off

Intraoperative QALAS protocol 1.3x1.3x1.3 mm3
TA: 7:51 PM: FIX Voxel size: 1.3×1.3×1.3 mmPAT: 2 Rel. SNR: 1.00 : qalas

Properties

Prio recon	Off
Load images to viewer	On
Inline movie	Off
Auto store images	On
Load images to stamp segments	Off
Load images to graphic segments	Off
Auto open inline display	Off
Auto close inline display	Off
Start measurement without further preparation	Off
Wait for user to start	Off
Start measurements	Single measurement

Routine

Slab group	1
Slabs	1
Dist. factor	50 %
Position	L1.5 A13.4 F0.5 mm
Orientation	Sagittal
Phase enc. dir.	A >> P
AutoAlign	Head > Basis
Phase oversampling	0 %
Slice oversampling	0.0 %
Slices per slab	144
FoV read	256 mm
FoV phase	101.0 %
Slice thickness	1.30 mm
TR	905.70 ms
TE	2.39 ms
Averages	1
Concatenations	1
Filter	Distortion Corr.(3D), Normalize
Coil elements	FL;FS;SP6-8

Contrast - Common

TR	905.70 ms
TE	2.39 ms
Flip angle	4 deg
Fat suppr.	None
Water suppr.	None

Contrast - Dynamic

Averages	1
Averaging mode	Long term
Reconstruction	Magn./Phase
Multiple series	Off

Resolution - Common

FoV read	256 mm
FoV phase	101.0 %
Slice thickness	1.30 mm
Base resolution	192
Phase resolution	100 %
Slice resolution	100 %
Phase partial Fourier	Off
Slice partial Fourier	Off
Interpolation	Off

Resolution - iPAT

PAT mode	GRAPPA
Accel. factor PE	2
Ref. lines PE	48
Accel. factor 3D	1
Reference scan mode	Integrated

Resolution - Filter Image

Image Filter	Off
Distortion Corr.	On
Mode	3D
Unfiltered images	On
Prescan Normalize	Off
Normalize	On
Unfiltered images	Off
B1 filter	Off

Resolution - Filter Rawdata

Raw filter	Off
Elliptical filter	Off

Geometry - Common

Slab group	1
Slabs	1
Dist. factor	50 %
Position	L1.5 A13.4 F0.5 mm
Orientation	Sagittal
Phase enc. dir.	A >> P
Slice oversampling	0.0 %
Slices per slab	144
FoV read	256 mm
FoV phase	101.0 %
Slice thickness	1.30 mm
TR	905.70 ms
Multi-slice mode	Sequential
Series	Interleaved
Concatenations	1

Geometry - AutoAlign

Slab group	1
Position	L1.5 A13.4 F0.5 mm
Orientation	Sagittal
Phase enc. dir.	A >> P
AutoAlign	Head > Basis
Initial Position	L1.5 A13.4 F0.5
L	1.5 mm
A	13.4 mm
F	0.5 mm
Initial Rotation	0.00 deg
Initial Orientation	Sagittal

Geometry - Navigator

System - Miscellaneous

Positioning mode	FIX
Table position	H
Table position	0 mm
MSMA	S - C - T
Sagittal	R >> L
Coronal	A >> P
Transversal	F >> H

System - Miscellaneous

Coil Combine Mode	Adaptive Combine
Save uncombined	Off
Matrix Optimization	Off
Coil Focus	Flat
AutoAlign	Head > Basis
Coil Select Mode	On - AutoCoilSelect

System - Adjustments

B0 Shim mode	Brain
B1 Shim mode	TrueForm
Adjust with body coil	Off
Confirm freq. adjustment	Off
Assume Dominant Fat	Off
Assume Silicone	Off
Adjustment Tolerance	Auto

System - Adjust Volume

Position	L1.5 A13.4 F0.5 mm
Orientation	Sagittal
Rotation	90.00 deg
F >> H	256 mm
A >> P	259 mm
R >> L	188 mm
Reset	Off

System - pTx Volumes

B1 Shim mode	TrueForm
Excitation	Slab-sel.

System - Tx/Rx

Frequency 1H	123.254412 MHz
Correction factor	1
Gain	Low
Img. Scale Cor.	1.000
Reset	Off
? Ref. amplitude 1H	0.000 V

Physio - Signal1

1st Signal/Mode	None
TR	905.70 ms
Concatenations	1

Physio - Cardiac

Fat suppr.	None
Dark blood	Off
FoV read	256 mm
FoV phase	101.0 %
Phase resolution	100 %

Physio - PACE

Resp. control	Off
Concatenations	1

Inline - Common

Subtract	Off
StdDev	Off
Save original images	On

Inline - MIP

MIP-Sag	Off
MIP-Cor	Off
MIP-Tra	Off
MIP-Time	Off

Inline - MIP

Save original images	On

Inline - Composing

Distortion Corr.	On
Mode	3D
Unfiltered images	On

Inline - MapIt

Save original images	On
MapIt	None
Flip angle	4 deg
TR	905.70 ms
TE	2.39 ms

Sequence - Part 1

Introduction	Off
Dimension	3D
Elliptical scanning	On
Reordering	Radial
Asymmetric echo	Off
Flow comp.	No
Multi-slice mode	Sequential
Echo spacing	5.7 ms
Bandwidth	400 Hz/Px

Sequence - Part 2

RF pulse type	Fast
Gradient mode	Fast
Excitation	Slab-sel.
RF spoiling	On
Incr. Gradient spoiling	On
Turbo factor	140

Sequence - Assistant

Mode	Off

Intraoperative QALAS protocol 1.3x1.3x1.3 mm3 with GD
TA: 7:51 PM: FIX Voxel size: 1.3×1.3×1.3 mmPAT: 2 Rel. SNR: 1.00 : qalas

Properties

Prio recon	Off
Load images to viewer	On
Inline movie	Off
Auto store images	On
Load images to stamp segments	Off
Load images to graphic segments	Off
Auto open inline display	Off
Auto close inline display	Off
Start measurement without further preparation	Off
Wait for user to start	Off
Start measurements	Single measurement

Routine

Slab group	1
Slabs	1
Dist. factor	50 %
Position	L1.5 A13.4 F0.5 mm
Orientation	Sagittal
Phase enc. dir.	A >> P
AutoAlign	Head > Basis
Phase oversampling	0 %
Slice oversampling	0.0 %
Slices per slab	144
FoV read	256 mm
FoV phase	101.0 %
Slice thickness	1.30 mm
TR	905.70 ms
TE	2.39 ms
Averages	1
Concatenations	1
Filter	Distortion Corr.(3D), Normalize
Coil elements	FL;FS;SP6-8

Contrast - Common

TR	905.70 ms
TE	2.39 ms
Flip angle	4 deg
Fat suppr.	None
Water suppr.	None

Contrast - Dynamic

Averages	1
Averaging mode	Long term
Reconstruction	Magn./Phase
Multiple series	Off

Resolution - Common

FoV read	256 mm
FoV phase	101.0 %
Slice thickness	1.30 mm
Base resolution	192
Phase resolution	100 %
Slice resolution	100 %
Phase partial Fourier	Off
Slice partial Fourier	Off
Interpolation	Off

Resolution - iPAT

PAT mode	GRAPPA
Accel. factor PE	2
Ref. lines PE	48
Accel. factor 3D	1
Reference scan mode	Integrated

Resolution - Filter Image

Image Filter	Off
Distortion Corr.	On
Mode	3D
Unfiltered images	On
Prescan Normalize	Off
Normalize	On
Unfiltered images	Off
B1 filter	Off

Resolution - Filter Rawdata

Raw filter	Off
Elliptical filter	Off

Geometry - Common

Slab group	1
Slabs	1
Dist. factor	50 %
Position	L1.5 A13.4 F0.5 mm
Orientation	Sagittal
Phase enc. dir.	A >> P
Slice oversampling	0.0 %
Slices per slab	144
FoV read	256 mm
FoV phase	101.0 %
Slice thickness	1.30 mm
TR	905.70 ms
Multi-slice mode	Sequential
Series	Interleaved
Concatenations	1

Geometry - AutoAlign

Slab group	1
Position	L1.5 A13.4 F0.5 mm
Orientation	Sagittal
Phase enc. dir.	A >> P
AutoAlign	Head > Basis
Initial Position	L1.5 A13.4 F0.5
L	1.5 mm
A	13.4 mm
F	0.5 mm
Initial Rotation	0.00 deg
Initial Orientation	Sagittal

Geometry - Navigator

System - Miscellaneous

Positioning mode	FIX
Table position	H
Table position	0 mm
MSMA	S - C - T
Sagittal	R >> L
Coronal	A >> P
Transversal	F >> H

System - Miscellaneous

Coil Combine Mode	Adaptive Combine
Save uncombined	Off
Matrix Optimization	Off
Coil Focus	Flat
AutoAlign	Head > Basis
Coil Select Mode	On - AutoCoilSelect

System - Adjustments

B0 Shim mode	Brain
B1 Shim mode	TrueForm
Adjust with body coil	Off
Confirm freq. adjustment	Off
Assume Dominant Fat	Off
Assume Silicone	Off
Adjustment Tolerance	Auto

System - Adjust Volume

Position	L1.5 A13.4 F0.5 mm
Orientation	Sagittal
Rotation	90.00 deg
F >> H	256 mm
A >> P	259 mm
R >> L	188 mm
Reset	Off

System - pTx Volumes

B1 Shim mode	TrueForm
Excitation	Slab-sel.

System - Tx/Rx

Frequency 1H	123.254412 MHz
Correction factor	1
Gain	Low
Img. Scale Cor.	1.000
Reset	Off
? Ref. amplitude 1H	0.000 V

Physio - Signal1

1st Signal/Mode	None
TR	905.70 ms
Concatenations	1

Physio - Cardiac

Fat suppr.	None
Dark blood	Off
FoV read	256 mm
FoV phase	101.0 %
Phase resolution	100 %

Physio - PACE

Resp. control	Off
Concatenations	1

Inline - Common

Subtract	Off
StdDev	Off
Save original images	On

Inline - MIP

MIP-Sag	Off
MIP-Cor	Off
MIP-Tra	Off
MIP-Time	Off

Inline - MIP

Save original images	On

Inline - Composing

Distortion Corr.	On
Mode	3D
Unfiltered images	On

Inline - MapIt

Save original images	On
MapIt	None
Flip angle	4 deg
TR	905.70 ms
TE	2.39 ms

Sequence - Part 1

Introduction	Off
Dimension	3D
Elliptical scanning	On
Reordering	Radial
Asymmetric echo	Off
Flow comp.	No
Multi-slice mode	Sequential
Echo spacing	5.7 ms
Bandwidth	400 Hz/Px

Sequence - Part 2

RF pulse type	Fast
Gradient mode	Fast
Excitation	Slab-sel.
RF spoiling	On
Incr. Gradient spoiling	On
Turbo factor	140

Sequence - Assistant

Mode	Off

Intraoperative B1map
TA: 0:12 PM: FIX Voxel size: 7.0×7.0×8.0 mmPAT: Off Rel. SNR: 1.00 : tfl

Properties

Prio recon	Off
Load images to viewer	On
Inline movie	Off
Auto store images	On
Load images to stamp segments	Off
Load images to graphic segments	Off
Auto open inline display	Off
Auto close inline display	Off
Start measurement without further preparation	Off
Wait for user to start	Off
Start measurements	Single measurement

Routine

Slice group	1
Slices	18
Dist. factor	100 %
Position	L1.5 A13.4 F0.5 mm
Orientation	Sagittal
Phase enc. dir.	A >> P
AutoAlign	Head > Basis
Phase oversampling	0 %
FoV read	450 mm
FoV phase	100.0 %
Slice thickness	8.0 mm
TR	5000.0 ms
TE	1.83 ms
Averages	1
Concatenations	1
Filter	None
Coil elements	HE1-4;NE1,2

Contrast - Common

TR	5000.0 ms
TE	1.83 ms
Magn. preparation	None
Flip angle	8 deg
Fat suppr.	None
Water suppr.	None

Contrast - Dynamic

Averages	1
Reconstruction	Magnitude
Measurements	1
Multiple series	Each measurement

Resolution - Common

FoV read	450 mm
FoV phase	100.0 %
Slice thickness	8.0 mm
Base resolution	64
Phase resolution	100 %
Phase partial Fourier	Off
Interpolation	Off

Resolution - iPAT

PAT mode	None

Resolution - Filter Image

Image Filter	Off
Distortion Corr.	Off
Prescan Normalize	Off
Normalize	Off
B1 filter	Off

Resolution - Filter Rawdata

Raw filter	Off
Elliptical filter	Off

Geometry - Common

Slice group	1
Slices	18
Dist. factor	100 %
Position	L1.5 A13.4 F0.5 mm
Orientation	Sagittal
Phase enc. dir.	A >> P
FoV read	450 mm
FoV phase	100.0 %
Slice thickness	8.0 mm
TR	5000.0 ms
Multi-slice mode	Interleaved
Series	Interleaved
Concatenations	1

Geometry - AutoAlign

Slice group	1
Position	L1.5 A13.4 F0.5 mm
Orientation	Sagittal
Phase enc. dir.	A >> P
AutoAlign	Head > Basis
Initial Position	L1.5 A13.4 F0.5
L	1.5 mm
A	13.4 mm
F	0.5 mm
Initial Rotation	0.00 deg
Initial Orientation	Sagittal

System - Miscellaneous

Positioning mode	FIX
Table position	H
Table position	0 mm
MSMA	S - C - T
Sagittal	R >> L
Coronal	A >> P
Transversal	F >> H
Coil Combine Mode	Sum of Squares
Save uncombined	Off
Matrix Optimization	Off
AutoAlign	Head > Basis
Coil Select Mode	Default

System - Adjustments

B0 Shim mode	Tune up
B1 Shim mode	TrueForm
Adjust with body coil	Off
Confirm freq. adjustment	Off
Assume Dominant Fat	Off
Assume Silicone	Off
Adjustment Tolerance	Auto

System - Adjust Volume

Position	Isocenter
Orientation	Transversal
Rotation	0.00 deg
A >> P	263 mm
R >> L	350 mm
F >> H	350 mm
Reset	Off

System - pTx Volumes

B1 Shim mode	TrueForm
Excitation	Slice-sel.

System - Tx/Rx

Frequency 1H	123.254412 MHz
Correction factor	1
Gain	High
Img. Scale Cor.	1.000
Reset	Off
? Ref. amplitude 1H	0.000 V

Inline - Common

Subtract	Off
Measurements	1
StdDev	Off
Save original images	On

Inline - MIP

MIP-Sag	Off
MIP-Cor	Off
MIP-Tra	Off
MIP-Time	Off
Save original images	On

Inline - Composing

Distortion Corr.	Off

Sequence - Part 1

Introduction	On
Dimension	2D
Asymmetric echo	Allowed
Flow comp.	No
Multi-slice mode	Interleaved
Echo spacing	4.1 ms
Bandwidth	490 Hz/Px

Sequence - Part 2

RF pulse type	Low SAR
Gradient mode	Normal
Excitation	Slice-sel.
RF spoiling	On
Turbo factor	64

Sequence - Assistant

Mode	Off

Gold standard T2map
TA: 16:07 PM: FIX Voxel size: 1.0×1.0×6.0 mmPAT: Off Rel. SNR: 1.00 : se

Properties

Prio recon	Off
Load images to viewer	On
Inline movie	Off
Auto store images	On
Load images to stamp segments	Off
Load images to graphic segments	Off
Auto open inline display	Off
Auto close inline display	Off
Start measurement without further preparation	Off
Wait for user to start	Off
Start measurements	Single measurement

Routine

Slice group	1
Slices	1
Dist. factor	100 %
Position	L1.5 A13.4 F0.5 mm
Orientation	Sagittal
Phase enc. dir.	A >> P
AutoAlign	Head > Basis
Phase oversampling	0 %
FoV read	256 mm
FoV phase	100.0 %
Slice thickness	6.0 mm
TR	5000.0 ms
TE 1	10.0 ms
TE 2	20.0 ms
TE 3	30.0 ms
TE 4	40.0 ms
TE 5	50.0 ms
TE 6	60.0 ms
TE 7	70.0 ms
TE 8	80.0 ms
TE 9	90.0 ms
TE 10	100 ms
TE 11	110.0 ms
TE 12	120.0 ms
TE 13	130.0 ms
TE 14	140.0 ms
TE 15	150.0 ms
TE 16	160.0 ms
TE 17	170.0 ms
TE 18	180.0 ms
TE 19	190.0 ms
TE 20	200 ms
TE 21	210.0 ms
TE 22	220.0 ms
TE 23	230.0 ms
TE 24	240.0 ms
TE 25	250.0 ms
TE 26	260.0 ms
TE 27	270.0 ms
TE 28	280.0 ms
TE 29	290.0 ms
TE 30	300 ms
TE 31	310.0 ms
TE 32	320.0 ms
Averages	1
Concatenations	1
Filter	None

Routine

Coil elements	HE1-4;NE1,2

Contrast - Common

TR	5000.0 ms
TE 1	10.0 ms
TE 2	20.0 ms
TE 3	30.0 ms
TE 4	40.0 ms
TE 5	50.0 ms
TE 6	60.0 ms
TE 7	70.0 ms
TE 8	80.0 ms
TE 9	90.0 ms
TE 10	100 ms
TE 11	110.0 ms
TE 12	120.0 ms
TE 13	130.0 ms
TE 14	140.0 ms
TE 15	150.0 ms
TE 16	160.0 ms
TE 17	170.0 ms
TE 18	180.0 ms
TE 19	190.0 ms
TE 20	200 ms
TE 21	210.0 ms
TE 22	220.0 ms
TE 23	230.0 ms
TE 24	240.0 ms
TE 25	250.0 ms
TE 26	260.0 ms
TE 27	270.0 ms
TE 28	280.0 ms
TE 29	290.0 ms
TE 30	300 ms
TE 31	310.0 ms
TE 32	320.0 ms
MTC	Off
Magn. preparation	None
Flip angle	180 deg
Fat suppr.	None
Water suppr.	None

Contrast - Dynamic

Averages	1
Averaging mode	Short term
Reconstruction	Magnitude
Measurements	1
Multiple series	Each measurement

Resolution - Common

FoV read	256 mm
FoV phase	100.0 %
Slice thickness	6.0 mm
Base resolution	256
Phase resolution	75 %
Phase partial Fourier	Off
Interpolation	Off

Resolution - iPAT

PAT mode	None

Resolution - Filter Image

Image Filter	Off
Distortion Corr.	Off
Prescan Normalize	Off
Normalize	Off
B1 filter	Off

Resolution - Filter Rawdata

Raw filter	Off
Elliptical filter	Off

Geometry - Common

Slice group	1
Slices	1
Dist. factor	100 %
Position	L1.5 A13.4 F0.5 mm
Orientation	Sagittal
Phase enc. dir.	A >> P
FoV read	256 mm
FoV phase	100.0 %
Slice thickness	6.0 mm
TR	5000.0 ms
Multi-slice mode	Interleaved
Series	Interleaved
Concatenations	1

Geometry - AutoAlign

Slice group	1
Position	L1.5 A13.4 F0.5 mm
Orientation	Sagittal
Phase enc. dir.	A >> P
AutoAlign	Head > Basis
Initial Position	L1.5 A13.4 F0.5
L	1.5 mm
A	13.4 mm
F	0.5 mm
Initial Rotation	0.00 deg
Initial Orientation	Sagittal

Geometry - Saturation

Fat suppr.	None
Water suppr.	None
Special sat.	None

System - Miscellaneous

Positioning mode	FIX
Table position	H
Table position	0 mm
MSMA	S - C - T
Sagittal	R >> L
Coronal	A >> P
Transversal	F >> H
Coil Combine Mode	Adaptive Combine
Save uncombined	Off
Matrix Optimization	Off
AutoAlign	Head > Basis
Coil Select Mode	Default

System - Adjustments

B0 Shim mode	Tune up
B1 Shim mode	TrueForm
Adjust with body coil	Off
Confirm freq. adjustment	Off
Assume Dominant Fat	Off
Assume Silicone	Off

System - Adjustments

Adjustment Tolerance	Auto

System - Adjust Volume

Position	Isocenter
Orientation	Transversal
Rotation	0.00 deg
A >> P	263 mm
R >> L	350 mm
F >> H	350 mm
Reset	Off

System - pTx Volumes

B1 Shim mode	TrueForm

System - Tx/Rx

Frequency 1H	123.254412 MHz
Correction factor	1
Gain	High
Img. Scale Cor.	1.000
Reset	Off
? Ref. amplitude 1H	0.000 V

Physio - Signal1

1st Signal/Mode	None
TR	5000.0 ms
Concatenations	1

Physio - Cardiac

Magn. preparation	None
Fat suppr.	None
Dark blood	Off
FoV read	256 mm
FoV phase	100.0 %
Phase resolution	75 %

Inline - Common

Subtract	Off
Measurements	1
StdDev	Off
Liver registration	Off
Save original images	On

Inline - MIP

MIP-Sag	Off
MIP-Cor	Off
MIP-Tra	Off
MIP-Time	Off
Save original images	On

Inline - Composing

Distortion Corr.	Off

Inline - MapIt

Save original images	On
MapIt	None
Flip angle	180 deg
Measurements	1
Contrasts	32
TR	5000.0 ms
TE 1	10.0 ms
TE 2	20.0 ms
TE 3	30.0 ms
TE 4	40.0 ms

Inline - MapIt

TE 5	50.0 ms
TE 6	60.0 ms
TE 7	70.0 ms
TE 8	80.0 ms
TE 9	90.0 ms
TE 10	100 ms
TE 11	110.0 ms
TE 12	120.0 ms
TE 13	130.0 ms
TE 14	140.0 ms
TE 15	150.0 ms
TE 16	160.0 ms
TE 17	170.0 ms
TE 18	180.0 ms
TE 19	190.0 ms
TE 20	200 ms
TE 21	210.0 ms
TE 22	220.0 ms
TE 23	230.0 ms
TE 24	240.0 ms
TE 25	250.0 ms
TE 26	260.0 ms
TE 27	270.0 ms
TE 28	280.0 ms
TE 29	290.0 ms
TE 30	300 ms
TE 31	310.0 ms
TE 32	320.0 ms

Sequence - Part 1

Introduction	On
Contrasts	32
Multi-slice mode	Interleaved
Bandwidth	227 Hz/Px

Sequence - Part 2

RF pulse type	Low SAR
Gradient mode	Fast

Sequence - Assistant

Mode	Off
Allowed delay	0 s

Gold standard T1map IR 35 ms

TA: 2:30 PM: FIX Voxel size: 1.0×1.0×6.0 mmPAT: Off Rel. SNR: 1.00 : tir

Properties

Prio recon	Off
Load images to viewer	On
Inline movie	Off
Auto store images	On
Load images to stamp segments	Off
Load images to graphic segments	Off
Auto open inline display	Off
Auto close inline display	Off
Start measurement without further preparation	Off
Wait for user to start	Off
Start measurements	Single measurement

Routine

Slice group	1
Slices	1
Dist. factor	50 %
Position	L1.5 A13.4 F0.5 mm
Orientation	Sagittal
Phase enc. dir.	A >> P
AutoAlign	Head > Basis
Phase oversampling	0 %
FoV read	256 mm
FoV phase	100.0 %
Slice thickness	6.0 mm
TR	4500.0 ms
TE	8.5 ms
Averages	1
Concatenations	1
Filter	None
Coil elements	HE1-4;NE1,2

Contrast - Common

TR	4500.0 ms
TE	8.5 ms
MTC	Off
Magn. preparation	Slice-sel. IR
TI	35 ms
Flip angle	180 deg
Fat suppr.	None
Water suppr.	None
Restore magn.	Off
Freeze suppressed tissue	Off

Contrast - Dynamic

Averages	1
Averaging mode	Short term
Reconstruction	Real
Measurements	1
Multiple series	Each measurement

Resolution - Common

FoV read	256 mm
FoV phase	100.0 %
Slice thickness	6.0 mm
Base resolution	256
Phase resolution	75 %
Phase partial Fourier	Off
Trajectory	Cartesian
Interpolation	Off

Resolution - iPAT

PAT mode	None

Resolution - Filter Image

Image Filter	Off
Distortion Corr.	Off
Prescan Normalize	Off
Normalize	Off
B1 filter	Off

Resolution - Filter Rawdata

Raw filter	Off
Elliptical filter	Off

Geometry - Common

Slice group	1
Slices	1
Dist. factor	50 %
Position	L1.5 A13.4 F0.5 mm
Orientation	Sagittal
Phase enc. dir.	A >> P
FoV read	256 mm
FoV phase	100.0 %
Slice thickness	6.0 mm
TR	4500.0 ms
Multi-slice mode	Interleaved
Series	Interleaved
Concatenations	1

Geometry - AutoAlign

Slice group	1
Position	L1.5 A13.4 F0.5 mm
Orientation	Sagittal
Phase enc. dir.	A >> P
AutoAlign	Head > Basis
Initial Position	L1.5 A13.4 F0.5
L	1.5 mm
A	13.4 mm
F	0.5 mm
Initial Rotation	0.00 deg
Initial Orientation	Sagittal

Geometry - Saturation

Fat suppr.	None
Water suppr.	None
Restore magn.	Off
Special sat.	None

Geometry - Navigator

System - Miscellaneous

Positioning mode	FIX
Table position	H
Table position	0 mm
MSMA	S - C - T
Sagittal	R >> L
Coronal	A >> P
Transversal	F >> H
Coil Combine Mode	Adaptive Combine
Save uncombined	Off
Matrix Optimization	Off

System - Miscellaneous

AutoAlign	Head > Basis
Coil Select Mode	Default

System - Adjustments

B0 Shim mode	Tune up
B1 Shim mode	TrueForm
Adjust with body coil	Off
Confirm freq. adjustment	Off
Assume Dominant Fat	Off
Assume Silicone	Off
Adjustment Tolerance	Auto

System - Adjust Volume

Position	Isocenter
Orientation	Transversal
Rotation	0.00 deg
A >> P	263 mm
R >> L	350 mm
F >> H	350 mm
Reset	Off

System - pTx Volumes

B1 Shim mode	TrueForm

System - Tx/Rx

Frequency 1H	123.254412 MHz
Correction factor	1
Gain	High
Img. Scale Cor.	1.000
Reset	Off
? Ref. amplitude 1H	0.000 V

Physio - Signal1

1st Signal/Mode	None
TR	4500.0 ms
Concatenations	1

Physio - Cardiac

Magn. preparation	Slice-sel. IR
TI	35 ms
Fat suppr.	None
Dark blood	Off
FoV read	256 mm
FoV phase	100.0 %
Phase resolution	75 %
Trajectory	Cartesian

Physio - PACE

Resp. control	Off
Concatenations	1

Inline - Common

Subtract	Off
Measurements	1
StdDev	Off
Save original images	On

Inline - MIP

MIP-Sag	Off
MIP-Cor	Off
MIP-Tra	Off
MIP-Time	Off
Save original images	On

Inline - Composing

Distortion Corr.	Off

Sequence - Part 1

Introduction	On
Dimension	2D
Compensate T2 decay	Off
Reduce Motion Sens.	Off
Contrasts	1
Flow comp.	No
Multi-slice mode	Interleaved
Free echo spacing	Off
Echo spacing	8.5 ms
Bandwidth	279 Hz/Px

Sequence - Part 2

Define	Turbo factor
Echo trains per slice	32
Phase correction	Automatic
Acoustic noise reduction	None
RF pulse type	Normal
Gradient mode	Fast
Hyperecho	Off
WARP	Off
Red. EC sensitivity	Off
Turbo factor	6

Sequence - Assistant

Mode	Off
Allowed delay	0 s

Gold standard T1map IR 75 ms

TA: 2:30 PM: FIX Voxel size: 1.0×1.0×6.0 mmPAT: Off Rel. SNR: 1.00 : tir

Properties

Prio recon	Off
Load images to viewer	On
Inline movie	Off
Auto store images	On
Load images to stamp segments	Off
Load images to graphic segments	Off
Auto open inline display	Off
Auto close inline display	Off
Start measurement without further preparation	Off
Wait for user to start	Off
Start measurements	Single measurement

Routine

Slice group	1
Slices	1
Dist. factor	50 %
Position	L1.5 A13.4 F0.5 mm
Orientation	Sagittal
Phase enc. dir.	A >> P
AutoAlign	Head > Basis
Phase oversampling	0 %
FoV read	256 mm
FoV phase	100.0 %
Slice thickness	6.0 mm
TR	4500.0 ms
TE	8.5 ms
Averages	1
Concatenations	1
Filter	None
Coil elements	HE1-4;NE1,2

Contrast - Common

TR	4500.0 ms
TE	8.5 ms
MTC	Off
Magn. preparation	Slice-sel. IR
TI	75 ms
Flip angle	180 deg
Fat suppr.	None
Water suppr.	None
Restore magn.	Off
Freeze suppressed tissue	Off

Contrast - Dynamic

Averages	1
Averaging mode	Short term
Reconstruction	Real
Measurements	1
Multiple series	Each measurement

Resolution - Common

FoV read	256 mm
FoV phase	100.0 %
Slice thickness	6.0 mm
Base resolution	256
Phase resolution	75 %
Phase partial Fourier	Off
Trajectory	Cartesian
Interpolation	Off

Resolution - iPAT

PAT mode	None

Resolution - Filter Image

Image Filter	Off
Distortion Corr.	Off
Prescan Normalize	Off
Normalize	Off
B1 filter	Off

Resolution - Filter Rawdata

Raw filter	Off
Elliptical filter	Off

Geometry - Common

Slice group	1
Slices	1
Dist. factor	50 %
Position	L1.5 A13.4 F0.5 mm
Orientation	Sagittal
Phase enc. dir.	A >> P
FoV read	256 mm
FoV phase	100.0 %
Slice thickness	6.0 mm
TR	4500.0 ms
Multi-slice mode	Interleaved
Series	Interleaved
Concatenations	1

Geometry - AutoAlign

Slice group	1
Position	L1.5 A13.4 F0.5 mm
Orientation	Sagittal
Phase enc. dir.	A >> P
AutoAlign	Head > Basis
Initial Position	L1.5 A13.4 F0.5
L	1.5 mm
A	13.4 mm
F	0.5 mm
Initial Rotation	0.00 deg
Initial Orientation	Sagittal

Geometry - Saturation

Fat suppr.	None
Water suppr.	None
Restore magn.	Off
Special sat.	None

Geometry - Navigator

System - Miscellaneous

Positioning mode	FIX
Table position	H
Table position	0 mm
MSMA	S - C - T
Sagittal	R >> L
Coronal	A >> P
Transversal	F >> H
Coil Combine Mode	Adaptive Combine
Save uncombined	Off
Matrix Optimization	Off

System - Miscellaneous

AutoAlign	Head > Basis
Coil Select Mode	Default

System - Adjustments

B0 Shim mode	Tune up
B1 Shim mode	TrueForm
Adjust with body coil	Off
Confirm freq. adjustment	Off
Assume Dominant Fat	Off
Assume Silicone	Off
Adjustment Tolerance	Auto

System - Adjust Volume

Position	Isocenter
Orientation	Transversal
Rotation	0.00 deg
A >> P	263 mm
R >> L	350 mm
F >> H	350 mm
Reset	Off

System - pTx Volumes

B1 Shim mode	TrueForm

System - Tx/Rx

Frequency 1H	123.254412 MHz
Correction factor	1
Gain	High
Img. Scale Cor.	1.000
Reset	Off
? Ref. amplitude 1H	0.000 V

Physio - Signal1

1st Signal/Mode	None
TR	4500.0 ms
Concatenations	1

Physio - Cardiac

Magn. preparation	Slice-sel. IR
TI	75 ms
Fat suppr.	None
Dark blood	Off
FoV read	256 mm
FoV phase	100.0 %
Phase resolution	75 %
Trajectory	Cartesian

Physio - PACE

Resp. control	Off
Concatenations	1

Inline - Common

Subtract	Off
Measurements	1
StdDev	Off
Save original images	On

Inline - MIP

MIP-Sag	Off
MIP-Cor	Off
MIP-Tra	Off
MIP-Time	Off
Save original images	On

Inline - Composing

Distortion Corr.	Off

Sequence - Part 1

Introduction	On
Dimension	2D
Compensate T2 decay	Off
Reduce Motion Sens.	Off
Contrasts	1
Flow comp.	No
Multi-slice mode	Interleaved
Free echo spacing	Off
Echo spacing	8.5 ms
Bandwidth	279 Hz/Px

Sequence - Part 2

Define	Turbo factor
Echo trains per slice	32
Phase correction	Automatic
Acoustic noise reduction	None
RF pulse type	Normal
Gradient mode	Fast
Hyperecho	Off
WARP	Off
Red. EC sensitivity	Off
Turbo factor	6

Sequence - Assistant

Mode	Off
Allowed delay	0 s

**Gold standard T1map IR 100 ms**
TA: 2:30 PM: FIX Voxel size: 1.0×1.0×6.0 mmPAT: Off Rel. SNR: 1.00 : tir

Properties

Prio recon	Off
Load images to viewer	On
Inline movie	Off
Auto store images	On
Load images to stamp segments	Off
Load images to graphic segments	Off
Auto open inline display	Off
Auto close inline display	Off
Start measurement without further preparation	Off
Wait for user to start	Off
Start measurements	Single measurement

Routine

Slice group	1
Slices	1
Dist. factor	50 %
Position	L1.5 A13.4 F0.5 mm
Orientation	Sagittal
Phase enc. dir.	A >> P
AutoAlign	Head > Basis
Phase oversampling	0 %
FoV read	256 mm
FoV phase	100.0 %
Slice thickness	6.0 mm
TR	4500.0 ms
TE	8.5 ms
Averages	1
Concatenations	1
Filter	None
Coil elements	HE1-4;NE1,2

Contrast - Common

TR	4500.0 ms
TE	8.5 ms
MTC	Off
Magn. preparation	Slice-sel. IR
TI	100 ms
Flip angle	180 deg
Fat suppr.	None
Water suppr.	None
Restore magn.	Off
Freeze suppressed tissue	Off

Contrast - Dynamic

Averages	1
Averaging mode	Short term
Reconstruction	Real
Measurements	1
Multiple series	Each measurement

Resolution - Common

FoV read	256 mm
FoV phase	100.0 %
Slice thickness	6.0 mm
Base resolution	256
Phase resolution	75 %
Phase partial Fourier	Off
Trajectory	Cartesian
Interpolation	Off

Resolution - iPAT

PAT mode	None

Resolution - Filter Image

Image Filter	Off
Distortion Corr.	Off
Prescan Normalize	Off
Normalize	Off
B1 filter	Off

Resolution - Filter Rawdata

Raw filter	Off
Elliptical filter	Off

Geometry - Common

Slice group	1
Slices	1
Dist. factor	50 %
Position	L1.5 A13.4 F0.5 mm
Orientation	Sagittal
Phase enc. dir.	A >> P
FoV read	256 mm
FoV phase	100.0 %
Slice thickness	6.0 mm
TR	4500.0 ms
Multi-slice mode	Interleaved
Series	Interleaved
Concatenations	1

Geometry - AutoAlign

Slice group	1
Position	L1.5 A13.4 F0.5 mm
Orientation	Sagittal
Phase enc. dir.	A >> P
AutoAlign	Head > Basis
Initial Position	L1.5 A13.4 F0.5
L	1.5 mm
A	13.4 mm
F	0.5 mm
Initial Rotation	0.00 deg
Initial Orientation	Sagittal

Geometry - Saturation

Fat suppr.	None
Water suppr.	None
Restore magn.	Off
Special sat.	None

Geometry - Navigator

System - Miscellaneous

Positioning mode	FIX
Table position	H
Table position	0 mm
MSMA	S - C - T
Sagittal	R >> L
Coronal	A >> P
Transversal	F >> H
Coil Combine Mode	Adaptive Combine
Save uncombined	Off
Matrix Optimization	Off

System - Miscellaneous

AutoAlign	Head > Basis
Coil Select Mode	Default

System - Adjustments

B0 Shim mode	Tune up
B1 Shim mode	TrueForm
Adjust with body coil	Off
Confirm freq. adjustment	Off
Assume Dominant Fat	Off
Assume Silicone	Off
Adjustment Tolerance	Auto

System - Adjust Volume

Position	Isocenter
Orientation	Transversal
Rotation	0.00 deg
A >> P	263 mm
R >> L	350 mm
F >> H	350 mm
Reset	Off

System - pTx Volumes

B1 Shim mode	TrueForm

System - Tx/Rx

Frequency 1H	123.254412 MHz
Correction factor	1
Gain	High
Img. Scale Cor.	1.000
Reset	Off
? Ref. amplitude 1H	0.000 V

Physio - Signal1

1st Signal/Mode	None
TR	4500.0 ms
Concatenations	1

Physio - Cardiac

Magn. preparation	Slice-sel. IR
TI	100 ms
Fat suppr.	None
Dark blood	Off
FoV read	256 mm
FoV phase	100.0 %
Phase resolution	75 %
Trajectory	Cartesian

Physio - PACE

Resp. control	Off
Concatenations	1

Inline - Common

Subtract	Off
Measurements	1
StdDev	Off
Save original images	On

Inline - MIP

MIP-Sag	Off
MIP-Cor	Off
MIP-Tra	Off
MIP-Time	Off
Save original images	On

Inline - Composing

Distortion Corr.	Off

Sequence - Part 1

Introduction	On
Dimension	2D
Compensate T2 decay	Off
Reduce Motion Sens.	Off
Contrasts	1
Flow comp.	No
Multi-slice mode	Interleaved
Free echo spacing	Off
Echo spacing	8.5 ms
Bandwidth	279 Hz/Px

Sequence - Part 2

Define	Turbo factor
Echo trains per slice	32
Phase correction	Automatic
Acoustic noise reduction	None
RF pulse type	Normal
Gradient mode	Fast
Hyperecho	Off
WARP	Off
Red. EC sensitivity	Off
Turbo factor	6

Sequence - Assistant

Mode	Off
Allowed delay	0 s

Gold standard T1map IR 125 ms
TA: 2:30 PM: FIX Voxel size: 1.0×1.0×6.0 mmPAT: Off Rel. SNR: 1.00 : tir

Properties

Prio recon	Off
Load images to viewer	On
Inline movie	Off
Auto store images	On
Load images to stamp segments	Off
Load images to graphic segments	Off
Auto open inline display	Off
Auto close inline display	Off
Start measurement without further preparation	Off
Wait for user to start	Off
Start measurements	Single measurement

Routine

Slice group	1
Slices	1
Dist. factor	50 %
Position	L1.5 A13.4 F0.5 mm
Orientation	Sagittal
Phase enc. dir.	A >> P
AutoAlign	Head > Basis
Phase oversampling	0 %
FoV read	256 mm
FoV phase	100.0 %
Slice thickness	6.0 mm
TR	4500.0 ms
TE	8.5 ms
Averages	1
Concatenations	1
Filter	None
Coil elements	HE1-4;NE1,2

Contrast - Common

TR	4500.0 ms
TE	8.5 ms
MTC	Off
Magn. preparation	Slice-sel. IR
TI	125 ms
Flip angle	180 deg
Fat suppr.	None
Water suppr.	None
Restore magn.	Off
Freeze suppressed tissue	Off

Contrast - Dynamic

Averages	1
Averaging mode	Short term
Reconstruction	Real
Measurements	1
Multiple series	Each measurement

Resolution - Common

FoV read	256 mm
FoV phase	100.0 %
Slice thickness	6.0 mm
Base resolution	256
Phase resolution	75 %
Phase partial Fourier	Off
Trajectory	Cartesian
Interpolation	Off

Resolution - iPAT

PAT mode	None

Resolution - Filter Image

Image Filter	Off
Distortion Corr.	Off
Prescan Normalize	Off
Normalize	Off
B1 filter	Off

Resolution - Filter Rawdata

Raw filter	Off
Elliptical filter	Off

Geometry - Common

Slice group	1
Slices	1
Dist. factor	50 %
Position	L1.5 A13.4 F0.5 mm
Orientation	Sagittal
Phase enc. dir.	A >> P
FoV read	256 mm
FoV phase	100.0 %
Slice thickness	6.0 mm
TR	4500.0 ms
Multi-slice mode	Interleaved
Series	Interleaved
Concatenations	1

Geometry - AutoAlign

Slice group	1
Position	L1.5 A13.4 F0.5 mm
Orientation	Sagittal
Phase enc. dir.	A >> P
AutoAlign	Head > Basis
Initial Position	L1.5 A13.4 F0.5
L	1.5 mm
A	13.4 mm
F	0.5 mm
Initial Rotation	0.00 deg
Initial Orientation	Sagittal

Geometry - Saturation

Fat suppr.	None
Water suppr.	None
Restore magn.	Off
Special sat.	None

Geometry - Navigator

System - Miscellaneous

Positioning mode	FIX
Table position	H
Table position	0 mm
MSMA	S - C - T
Sagittal	R >> L
Coronal	A >> P
Transversal	F >> H
Coil Combine Mode	Adaptive Combine
Save uncombined	Off
Matrix Optimization	Off

System - Miscellaneous

AutoAlign	Head > Basis
Coil Select Mode	Default

System - Adjustments

B0 Shim mode	Tune up
B1 Shim mode	TrueForm
Adjust with body coil	Off
Confirm freq. adjustment	Off
Assume Dominant Fat	Off
Assume Silicone	Off
Adjustment Tolerance	Auto

System - Adjust Volume

Position	Isocenter
Orientation	Transversal
Rotation	0.00 deg
A >> P	263 mm
R >> L	350 mm
F >> H	350 mm
Reset	Off

System - pTx Volumes

B1 Shim mode	TrueForm

System - Tx/Rx

Frequency 1H	123.254412 MHz
Correction factor	1
Gain	High
Img. Scale Cor.	1.000
Reset	Off
? Ref. amplitude 1H	0.000 V

Physio - Signal1

1st Signal/Mode	None
TR	4500.0 ms
Concatenations	1

Physio - Cardiac

Magn. preparation	Slice-sel. IR
TI	125 ms
Fat suppr.	None
Dark blood	Off
FoV read	256 mm
FoV phase	100.0 %
Phase resolution	75 %
Trajectory	Cartesian

Physio - PACE

Resp. control	Off
Concatenations	1

Inline - Common

Subtract	Off
Measurements	1
StdDev	Off
Save original images	On

Inline - MIP

MIP-Sag	Off
MIP-Cor	Off
MIP-Tra	Off
MIP-Time	Off
Save original images	On

Inline - Composing

Distortion Corr.	Off

Sequence - Part 1

Introduction	On
Dimension	2D
Compensate T2 decay	Off
Reduce Motion Sens.	Off
Contrasts	1
Flow comp.	No
Multi-slice mode	Interleaved
Free echo spacing	Off
Echo spacing	8.5 ms
Bandwidth	279 Hz/Px

Sequence - Part 2

Define	Turbo factor
Echo trains per slice	32
Phase correction	Automatic
Acoustic noise reduction	None
RF pulse type	Normal
Gradient mode	Fast
Hyperecho	Off
WARP	Off
Red. EC sensitivity	Off
Turbo factor	6

Sequence - Assistant

Mode	Off
Allowed delay	0 s

Gold standard T1map IR 150 ms
TA: 2:30 PM: FIX Voxel size: 1.0×1.0×6.0 mmPAT: Off Rel. SNR: 1.00 : tir

Properties

Prio recon	Off
Load images to viewer	On
Inline movie	Off
Auto store images	On
Load images to stamp segments	Off
Load images to graphic segments	Off
Auto open inline display	Off
Auto close inline display	Off
Start measurement without further preparation	Off
Wait for user to start	Off
Start measurements	Single measurement

Routine

Slice group	1
Slices	1
Dist. factor	50 %
Position	L1.5 A13.4 F0.5 mm
Orientation	Sagittal
Phase enc. dir.	A >> P
AutoAlign	Head > Basis
Phase oversampling	0 %
FoV read	256 mm
FoV phase	100.0 %
Slice thickness	6.0 mm
TR	4500.0 ms
TE	8.5 ms
Averages	1
Concatenations	1
Filter	None
Coil elements	HE1-4;NE1,2

Contrast - Common

TR	4500.0 ms
TE	8.5 ms
MTC	Off
Magn. preparation	Slice-sel. IR
TI	150 ms
Flip angle	180 deg
Fat suppr.	None
Water suppr.	None
Restore magn.	Off
Freeze suppressed tissue	Off

Contrast - Dynamic

Averages	1
Averaging mode	Short term
Reconstruction	Real
Measurements	1
Multiple series	Each measurement

Resolution - Common

FoV read	256 mm
FoV phase	100.0 %
Slice thickness	6.0 mm
Base resolution	256
Phase resolution	75 %
Phase partial Fourier	Off
Trajectory	Cartesian
Interpolation	Off

Resolution - iPAT

PAT mode	None

Resolution - Filter Image

Image Filter	Off
Distortion Corr.	Off
Prescan Normalize	Off
Normalize	Off
B1 filter	Off

Resolution - Filter Rawdata

Raw filter	Off
Elliptical filter	Off

Geometry - Common

Slice group	1
Slices	1
Dist. factor	50 %
Position	L1.5 A13.4 F0.5 mm
Orientation	Sagittal
Phase enc. dir.	A >> P
FoV read	256 mm
FoV phase	100.0 %
Slice thickness	6.0 mm
TR	4500.0 ms
Multi-slice mode	Interleaved
Series	Interleaved
Concatenations	1

Geometry - AutoAlign

Slice group	1
Position	L1.5 A13.4 F0.5 mm
Orientation	Sagittal
Phase enc. dir.	A >> P
AutoAlign	Head > Basis
Initial Position	L1.5 A13.4 F0.5
L	1.5 mm
A	13.4 mm
F	0.5 mm
Initial Rotation	0.00 deg
Initial Orientation	Sagittal

Geometry - Saturation

Fat suppr.	None
Water suppr.	None
Restore magn.	Off
Special sat.	None

Geometry - Navigator

System - Miscellaneous

Positioning mode	FIX
Table position	H
Table position	0 mm
MSMA	S - C - T
Sagittal	R >> L
Coronal	A >> P
Transversal	F >> H
Coil Combine Mode	Adaptive Combine
Save uncombined	Off
Matrix Optimization	Off

System - Miscellaneous

AutoAlign	Head > Basis
Coil Select Mode	Default

System - Adjustments

B0 Shim mode	Tune up
B1 Shim mode	TrueForm
Adjust with body coil	Off
Confirm freq. adjustment	Off
Assume Dominant Fat	Off
Assume Silicone	Off
Adjustment Tolerance	Auto

System - Adjust Volume

Position	Isocenter
Orientation	Transversal
Rotation	0.00 deg
A >> P	263 mm
R >> L	350 mm
F >> H	350 mm
Reset	Off

System - pTx Volumes

B1 Shim mode	TrueForm

System - Tx/Rx

Frequency 1H	123.254412 MHz
Correction factor	1
Gain	High
Img. Scale Cor.	1.000
Reset	Off
? Ref. amplitude 1H	0.000 V

Physio - Signal1

1st Signal/Mode	None
TR	4500.0 ms
Concatenations	1

Physio - Cardiac

Magn. preparation	Slice-sel. IR
TI	150 ms
Fat suppr.	None
Dark blood	Off
FoV read	256 mm
FoV phase	100.0 %
Phase resolution	75 %
Trajectory	Cartesian

Physio - PACE

Resp. control	Off
Concatenations	1

Inline - Common

Subtract	Off
Measurements	1
StdDev	Off
Save original images	On

Inline - MIP

MIP-Sag	Off
MIP-Cor	Off
MIP-Tra	Off
MIP-Time	Off
Save original images	On

Inline - Composing

Distortion Corr.	Off

Sequence - Part 1

Introduction	On
Dimension	2D
Compensate T2 decay	Off
Reduce Motion Sens.	Off
Contrasts	1
Flow comp.	No
Multi-slice mode	Interleaved
Free echo spacing	Off
Echo spacing	8.5 ms
Bandwidth	279 Hz/Px

Sequence - Part 2

Define	Turbo factor
Echo trains per slice	32
Phase correction	Automatic
Acoustic noise reduction	None
RF pulse type	Normal
Gradient mode	Fast
Hyperecho	Off
WARP	Off
Red. EC sensitivity	Off
Turbo factor	6

Sequence - Assistant

Mode	Off
Allowed delay	0 s

Gold standard T1map IR 250 ms
TA: 2:30 PM: FIX Voxel size: 1.0×1.0×6.0 mmPAT: Off Rel. SNR: 1.00 : tir

Properties

Prio recon	Off
Load images to viewer	On
Inline movie	Off
Auto store images	On
Load images to stamp segments	Off
Load images to graphic segments	Off
Auto open inline display	Off
Auto close inline display	Off
Start measurement without further preparation	Off
Wait for user to start	Off
Start measurements	Single measurement

Routine

Slice group	1
Slices	1
Dist. factor	50 %
Position	L1.5 A13.4 F0.5 mm
Orientation	Sagittal
Phase enc. dir.	A >> P
AutoAlign	Head > Basis
Phase oversampling	0 %
FoV read	256 mm
FoV phase	100.0 %
Slice thickness	6.0 mm
TR	4500.0 ms
TE	8.5 ms
Averages	1
Concatenations	1
Filter	None
Coil elements	HE1-4;NE1,2

Contrast - Common

TR	4500.0 ms
TE	8.5 ms
MTC	Off
Magn. preparation	Slice-sel. IR
TI	250 ms
Flip angle	180 deg
Fat suppr.	None
Water suppr.	None
Restore magn.	Off
Freeze suppressed tissue	Off

Contrast - Dynamic

Averages	1
Averaging mode	Short term
Reconstruction	Real
Measurements	1
Multiple series	Each measurement

Resolution - Common

FoV read	256 mm
FoV phase	100.0 %
Slice thickness	6.0 mm
Base resolution	256
Phase resolution	75 %
Phase partial Fourier	Off
Trajectory	Cartesian
Interpolation	Off

Resolution - iPAT

PAT mode	None

Resolution - Filter Image

Image Filter	Off
Distortion Corr.	Off
Prescan Normalize	Off
Normalize	Off
B1 filter	Off

Resolution - Filter Rawdata

Raw filter	Off
Elliptical filter	Off

Geometry - Common

Slice group	1
Slices	1
Dist. factor	50 %
Position	L1.5 A13.4 F0.5 mm
Orientation	Sagittal
Phase enc. dir.	A >> P
FoV read	256 mm
FoV phase	100.0 %
Slice thickness	6.0 mm
TR	4500.0 ms
Multi-slice mode	Interleaved
Series	Interleaved
Concatenations	1

Geometry - AutoAlign

Slice group	1
Position	L1.5 A13.4 F0.5 mm
Orientation	Sagittal
Phase enc. dir.	A >> P
AutoAlign	Head > Basis
Initial Position	L1.5 A13.4 F0.5
L	1.5 mm
A	13.4 mm
F	0.5 mm
Initial Rotation	0.00 deg
Initial Orientation	Sagittal

Geometry - Saturation

Fat suppr.	None
Water suppr.	None
Restore magn.	Off
Special sat.	None

Geometry - Navigator

System - Miscellaneous

Positioning mode	FIX
Table position	H
Table position	0 mm
MSMA	S - C - T
Sagittal	R >> L
Coronal	A >> P
Transversal	F >> H
Coil Combine Mode	Adaptive Combine
Save uncombined	Off
Matrix Optimization	Off

System - Miscellaneous

AutoAlign	Head > Basis
Coil Select Mode	Default

System - Adjustments

B0 Shim mode	Tune up
B1 Shim mode	TrueForm
Adjust with body coil	Off
Confirm freq. adjustment	Off
Assume Dominant Fat	Off
Assume Silicone	Off
Adjustment Tolerance	Auto

System - Adjust Volume

Position	Isocenter
Orientation	Transversal
Rotation	0.00 deg
A >> P	263 mm
R >> L	350 mm
F >> H	350 mm
Reset	Off

System - pTx Volumes

B1 Shim mode	TrueForm

System - Tx/Rx

Frequency 1H	123.254412 MHz
Correction factor	1
Gain	High
Img. Scale Cor.	1.000
Reset	Off
? Ref. amplitude 1H	0.000 V

Physio - Signal1

1st Signal/Mode	None
TR	4500.0 ms
Concatenations	1

Physio - Cardiac

Magn. preparation	Slice-sel. IR
TI	250 ms
Fat suppr.	None
Dark blood	Off
FoV read	256 mm
FoV phase	100.0 %
Phase resolution	75 %
Trajectory	Cartesian

Physio - PACE

Resp. control	Off
Concatenations	1

Inline - Common

Subtract	Off
Measurements	1
StdDev	Off
Save original images	On

Inline - MIP

MIP-Sag	Off
MIP-Cor	Off
MIP-Tra	Off
MIP-Time	Off
Save original images	On

Inline - Composing

Distortion Corr.	Off

Sequence - Part 1

Introduction	On
Dimension	2D
Compensate T2 decay	Off
Reduce Motion Sens.	Off
Contrasts	1
Flow comp.	No
Multi-slice mode	Interleaved
Free echo spacing	Off
Echo spacing	8.5 ms
Bandwidth	279 Hz/Px

Sequence - Part 2

Define	Turbo factor
Echo trains per slice	32
Phase correction	Automatic
Acoustic noise reduction	None
RF pulse type	Normal
Gradient mode	Fast
Hyperecho	Off
WARP	Off
Red. EC sensitivity	Off
Turbo factor	6

Sequence - Assistant

Mode	Off
Allowed delay	0 s

Gold standard T1map IR 1000 ms
TA: 2:30 PM: FIX Voxel size: 1.0×1.0×6.0 mmPAT: Off Rel. SNR: 1.00 : tir

Properties

Prio recon	Off
Load images to viewer	On
Inline movie	Off
Auto store images	On
Load images to stamp segments	Off
Load images to graphic segments	Off
Auto open inline display	Off
Auto close inline display	Off
Start measurement without further preparation	Off
Wait for user to start	Off
Start measurements	Single measurement

Routine

Slice group	1
Slices	1
Dist. factor	50 %
Position	L1.5 A13.4 F0.5 mm
Orientation	Sagittal
Phase enc. dir.	A >> P
AutoAlign	Head > Basis
Phase oversampling	0 %
FoV read	256 mm
FoV phase	100.0 %
Slice thickness	6.0 mm
TR	4500.0 ms
TE	8.5 ms
Averages	1
Concatenations	1
Filter	None
Coil elements	HE1-4;NE1,2

Contrast - Common

TR	4500.0 ms
TE	8.5 ms
MTC	Off
Magn. preparation	Slice-sel. IR
TI	1000 ms
Flip angle	180 deg
Fat suppr.	None
Water suppr.	None
Restore magn.	Off
Freeze suppressed tissue	Off

Contrast - Dynamic

Averages	1
Averaging mode	Short term
Reconstruction	Real
Measurements	1
Multiple series	Each measurement

Resolution - Common

FoV read	256 mm
FoV phase	100.0 %
Slice thickness	6.0 mm
Base resolution	256
Phase resolution	75 %
Phase partial Fourier	Off
Trajectory	Cartesian
Interpolation	Off

Resolution - iPAT

PAT mode	None

Resolution - Filter Image

Image Filter	Off
Distortion Corr.	Off
Prescan Normalize	Off
Normalize	Off
B1 filter	Off

Resolution - Filter Rawdata

Raw filter	Off
Elliptical filter	Off

Geometry - Common

Slice group	1
Slices	1
Dist. factor	50 %
Position	L1.5 A13.4 F0.5 mm
Orientation	Sagittal
Phase enc. dir.	A >> P
FoV read	256 mm
FoV phase	100.0 %
Slice thickness	6.0 mm
TR	4500.0 ms
Multi-slice mode	Interleaved
Series	Interleaved
Concatenations	1

Geometry - AutoAlign

Slice group	1
Position	L1.5 A13.4 F0.5 mm
Orientation	Sagittal
Phase enc. dir.	A >> P
AutoAlign	Head > Basis
Initial Position	L1.5 A13.4 F0.5
L	1.5 mm
A	13.4 mm
F	0.5 mm
Initial Rotation	0.00 deg
Initial Orientation	Sagittal

Geometry - Saturation

Fat suppr.	None
Water suppr.	None
Restore magn.	Off
Special sat.	None

Geometry - Navigator

System - Miscellaneous

Positioning mode	FIX
Table position	H
Table position	0 mm
MSMA	S - C - T
Sagittal	R >> L
Coronal	A >> P
Transversal	F >> H
Coil Combine Mode	Adaptive Combine
Save uncombined	Off
Matrix Optimization	Off

System - Miscellaneous

AutoAlign	Head > Basis
Coil Select Mode	Default

System - Adjustments

B0 Shim mode	Tune up
B1 Shim mode	TrueForm
Adjust with body coil	Off
Confirm freq. adjustment	Off
Assume Dominant Fat	Off
Assume Silicone	Off
Adjustment Tolerance	Auto

System - Adjust Volume

Position	Isocenter
Orientation	Transversal
Rotation	0.00 deg
A >> P	263 mm
R >> L	350 mm
F >> H	350 mm
Reset	Off

System - pTx Volumes

B1 Shim mode	TrueForm

System - Tx/Rx

Frequency 1H	123.254412 MHz
Correction factor	1
Gain	High
Img. Scale Cor.	1.000
Reset	Off
? Ref. amplitude 1H	0.000 V

Physio - Signal1

1st Signal/Mode	None
TR	4500.0 ms
Concatenations	1

Physio - Cardiac

Magn. preparation	Slice-sel. IR
TI	1000 ms
Fat suppr.	None
Dark blood	Off
FoV read	256 mm
FoV phase	100.0 %
Phase resolution	75 %
Trajectory	Cartesian

Physio - PACE

Resp. control	Off
Concatenations	1

Inline - Common

Subtract	Off
Measurements	1
StdDev	Off
Save original images	On

Inline - MIP

MIP-Sag	Off
MIP-Cor	Off
MIP-Tra	Off
MIP-Time	Off
Save original images	On

Inline - Composing

Distortion Corr.	Off

Sequence - Part 1

Introduction	On
Dimension	2D
Compensate T2 decay	Off
Reduce Motion Sens.	Off
Contrasts	1
Flow comp.	No
Multi-slice mode	Interleaved
Free echo spacing	Off
Echo spacing	8.5 ms
Bandwidth	279 Hz/Px

Sequence - Part 2

Define	Turbo factor
Echo trains per slice	32
Phase correction	Automatic
Acoustic noise reduction	None
RF pulse type	Normal
Gradient mode	Fast
Hyperecho	Off
WARP	Off
Red. EC sensitivity	Off
Turbo factor	6

Sequence - Assistant

Mode	Off
Allowed delay	0 s

Gold standard T1map IR 1500 ms
TA: 2:30 PM: FIX Voxel size: 1.0×1.0×6.0 mmPAT: Off Rel. SNR: 1.00 : tir

Properties

Prio recon	Off
Load images to viewer	On
Inline movie	Off
Auto store images	On
Load images to stamp segments	Off
Load images to graphic segments	Off
Auto open inline display	Off
Auto close inline display	Off
Start measurement without further preparation	Off
Wait for user to start	Off
Start measurements	Single measurement

Routine

Slice group	1
Slices	1
Dist. factor	50 %
Position	L1.5 A13.4 F0.5 mm
Orientation	Sagittal
Phase enc. dir.	A >> P
AutoAlign	Head > Basis
Phase oversampling	0 %
FoV read	256 mm
FoV phase	100.0 %
Slice thickness	6.0 mm
TR	4500.0 ms
TE	8.5 ms
Averages	1
Concatenations	1
Filter	None
Coil elements	HE1-4;NE1,2

Contrast - Common

TR	4500.0 ms
TE	8.5 ms
MTC	Off
Magn. preparation	Slice-sel. IR
TI	1500 ms
Flip angle	180 deg
Fat suppr.	None
Water suppr.	None
Restore magn.	Off
Freeze suppressed tissue	Off

Contrast - Dynamic

Averages	1
Averaging mode	Short term
Reconstruction	Real
Measurements	1
Multiple series	Each measurement

Resolution - Common

FoV read	256 mm
FoV phase	100.0 %
Slice thickness	6.0 mm
Base resolution	256
Phase resolution	75 %
Phase partial Fourier	Off
Trajectory	Cartesian
Interpolation	Off

Resolution - iPAT

PAT mode	None

Resolution - Filter Image

Image Filter	Off
Distortion Corr.	Off
Prescan Normalize	Off
Normalize	Off
B1 filter	Off

Resolution - Filter Rawdata

Raw filter	Off
Elliptical filter	Off

Geometry - Common

Slice group	1
Slices	1
Dist. factor	50 %
Position	L1.5 A13.4 F0.5 mm
Orientation	Sagittal
Phase enc. dir.	A >> P
FoV read	256 mm
FoV phase	100.0 %
Slice thickness	6.0 mm
TR	4500.0 ms
Multi-slice mode	Interleaved
Series	Interleaved
Concatenations	1

Geometry - AutoAlign

Slice group	1
Position	L1.5 A13.4 F0.5 mm
Orientation	Sagittal
Phase enc. dir.	A >> P
AutoAlign	Head > Basis
Initial Position	L1.5 A13.4 F0.5
L	1.5 mm
A	13.4 mm
F	0.5 mm
Initial Rotation	0.00 deg
Initial Orientation	Sagittal

Geometry - Saturation

Fat suppr.	None
Water suppr.	None
Restore magn.	Off
Special sat.	None

Geometry - Navigator

System - Miscellaneous

Positioning mode	FIX
Table position	H
Table position	0 mm
MSMA	S - C - T
Sagittal	R >> L
Coronal	A >> P
Transversal	F >> H
Coil Combine Mode	Adaptive Combine
Save uncombined	Off
Matrix Optimization	Off

System - Miscellaneous

AutoAlign	Head > Basis
Coil Select Mode	Default

System - Adjustments

B0 Shim mode	Tune up
B1 Shim mode	TrueForm
Adjust with body coil	Off
Confirm freq. adjustment	Off
Assume Dominant Fat	Off
Assume Silicone	Off
Adjustment Tolerance	Auto

System - Adjust Volume

Position	Isocenter
Orientation	Transversal
Rotation	0.00 deg
A >> P	263 mm
R >> L	350 mm
F >> H	350 mm
Reset	Off

System - pTx Volumes

B1 Shim mode	TrueForm

System - Tx/Rx

Frequency 1H	123.254412 MHz
Correction factor	1
Gain	High
Img. Scale Cor.	1.000
Reset	Off
? Ref. amplitude 1H	0.000 V

Physio - Signal1

1st Signal/Mode	None
TR	4500.0 ms
Concatenations	1

Physio - Cardiac

Magn. preparation	Slice-sel. IR
TI	1500 ms
Fat suppr.	None
Dark blood	Off
FoV read	256 mm
FoV phase	100.0 %
Phase resolution	75 %
Trajectory	Cartesian

Physio - PACE

Resp. control	Off
Concatenations	1

Inline - Common

Subtract	Off
Measurements	1
StdDev	Off
Save original images	On

Inline - MIP

MIP-Sag	Off
MIP-Cor	Off
MIP-Tra	Off
MIP-Time	Off
Save original images	On

Inline - Composing

Distortion Corr.	Off

Sequence - Part 1

Introduction	On
Dimension	2D
Compensate T2 decay	Off
Reduce Motion Sens.	Off
Contrasts	1
Flow comp.	No
Multi-slice mode	Interleaved
Free echo spacing	Off
Echo spacing	8.5 ms
Bandwidth	279 Hz/Px

Sequence - Part 2

Define	Turbo factor
Echo trains per slice	32
Phase correction	Automatic
Acoustic noise reduction	None
RF pulse type	Normal
Gradient mode	Fast
Hyperecho	Off
WARP	Off
Red. EC sensitivity	Off
Turbo factor	6

Sequence - Assistant

Mode	Off
Allowed delay	0 s

Gold standard T1map IR 2000 ms
TA: 2:30 PM: FIX Voxel size: 1.0×1.0×6.0 mmPAT: Off Rel. SNR: 1.00 : tir

Properties

Prio recon	Off
Load images to viewer	On
Inline movie	Off
Auto store images	On
Load images to stamp segments	Off
Load images to graphic segments	Off
Auto open inline display	Off
Auto close inline display	Off
Start measurement without further preparation	Off
Wait for user to start	Off
Start measurements	Single measurement

Routine

Slice group	1
Slices	1
Dist. factor	50 %
Position	L1.5 A13.4 F0.5 mm
Orientation	Sagittal
Phase enc. dir.	A >> P
AutoAlign	Head > Basis
Phase oversampling	0 %
FoV read	256 mm
FoV phase	100.0 %
Slice thickness	6.0 mm
TR	4500.0 ms
TE	8.5 ms
Averages	1
Concatenations	1
Filter	None
Coil elements	HE1-4;NE1,2

Contrast - Common

TR	4500.0 ms
TE	8.5 ms
MTC	Off
Magn. preparation	Slice-sel. IR
TI	2000 ms
Flip angle	180 deg
Fat suppr.	None
Water suppr.	None
Restore magn.	Off
Freeze suppressed tissue	Off

Contrast - Dynamic

Averages	1
Averaging mode	Short term
Reconstruction	Real
Measurements	1
Multiple series	Each measurement

Resolution - Common

FoV read	256 mm
FoV phase	100.0 %
Slice thickness	6.0 mm
Base resolution	256
Phase resolution	75 %
Phase partial Fourier	Off
Trajectory	Cartesian
Interpolation	Off

Resolution - iPAT

PAT mode	None

Resolution - Filter Image

Image Filter	Off
Distortion Corr.	Off
Prescan Normalize	Off
Normalize	Off
B1 filter	Off

Resolution - Filter Rawdata

Raw filter	Off
Elliptical filter	Off

Geometry - Common

Slice group	1
Slices	1
Dist. factor	50 %
Position	L1.5 A13.4 F0.5 mm
Orientation	Sagittal
Phase enc. dir.	A >> P
FoV read	256 mm
FoV phase	100.0 %
Slice thickness	6.0 mm
TR	4500.0 ms
Multi-slice mode	Interleaved
Series	Interleaved
Concatenations	1

Geometry - AutoAlign

Slice group	1
Position	L1.5 A13.4 F0.5 mm
Orientation	Sagittal
Phase enc. dir.	A >> P
AutoAlign	Head > Basis
Initial Position	L1.5 A13.4 F0.5
L	1.5 mm
A	13.4 mm
F	0.5 mm
Initial Rotation	0.00 deg
Initial Orientation	Sagittal

Geometry - Saturation

Fat suppr.	None
Water suppr.	None
Restore magn.	Off
Special sat.	None

Geometry - Navigator

System - Miscellaneous

Positioning mode	FIX
Table position	H
Table position	0 mm
MSMA	S - C - T
Sagittal	R >> L
Coronal	A >> P
Transversal	F >> H
Coil Combine Mode	Adaptive Combine
Save uncombined	Off
Matrix Optimization	Off

System - Miscellaneous

AutoAlign	Head > Basis
Coil Select Mode	Default

System - Adjustments

B0 Shim mode	Tune up
B1 Shim mode	TrueForm
Adjust with body coil	Off
Confirm freq. adjustment	Off
Assume Dominant Fat	Off
Assume Silicone	Off
Adjustment Tolerance	Auto

System - Adjust Volume

Position	Isocenter
Orientation	Transversal
Rotation	0.00 deg
A >> P	263 mm
R >> L	350 mm
F >> H	350 mm
Reset	Off

System - pTx Volumes

B1 Shim mode	TrueForm

System - Tx/Rx

Frequency 1H	123.254412 MHz
Correction factor	1
Gain	High
Img. Scale Cor.	1.000
Reset	Off
? Ref. amplitude 1H	0.000 V

Physio - Signal1

1st Signal/Mode	None
TR	4500.0 ms
Concatenations	1

Physio - Cardiac

Magn. preparation	Slice-sel. IR
TI	2000 ms
Fat suppr.	None
Dark blood	Off
FoV read	256 mm
FoV phase	100.0 %
Phase resolution	75 %
Trajectory	Cartesian

Physio - PACE

Resp. control	Off
Concatenations	1

Inline - Common

Subtract	Off
Measurements	1
StdDev	Off
Save original images	On

Inline - MIP

MIP-Sag	Off
MIP-Cor	Off
MIP-Tra	Off
MIP-Time	Off
Save original images	On

Inline - Composing

Distortion Corr.	Off

Sequence - Part 1

Introduction	On
Dimension	2D
Compensate T2 decay	Off
Reduce Motion Sens.	Off
Contrasts	1
Flow comp.	No
Multi-slice mode	Interleaved
Free echo spacing	Off
Echo spacing	8.5 ms
Bandwidth	279 Hz/Px

Sequence - Part 2

Define	Turbo factor
Echo trains per slice	32
Phase correction	Automatic
Acoustic noise reduction	None
RF pulse type	Normal
Gradient mode	Fast
Hyperecho	Off
WARP	Off
Red. EC sensitivity	Off
Turbo factor	6

Sequence - Assistant

Mode	Off
Allowed delay	0 s

Gold standard T1map IR 3000 ms
TA: 2:30 PM: FIX Voxel size: 1.0×1.0×6.0 mmPAT: Off Rel. SNR: 1.00 : tir

Properties

Prio recon	Off
Load images to viewer	On
Inline movie	Off
Auto store images	On
Load images to stamp segments	Off
Load images to graphic segments	Off
Auto open inline display	Off
Auto close inline display	Off
Start measurement without further preparation	Off
Wait for user to start	Off
Start measurements	Single measurement

Routine

Slice group	1
Slices	1
Dist. factor	50 %
Position	L1.5 A13.4 F0.5 mm
Orientation	Sagittal
Phase enc. dir.	A >> P
AutoAlign	Head > Basis
Phase oversampling	0 %
FoV read	256 mm
FoV phase	100.0 %
Slice thickness	6.0 mm
TR	4500.0 ms
TE	8.5 ms
Averages	1
Concatenations	1
Filter	None
Coil elements	HE1-4;NE1,2

Contrast - Common

TR	4500.0 ms
TE	8.5 ms
MTC	Off
Magn. preparation	Slice-sel. IR
TI	3000 ms
Flip angle	180 deg
Fat suppr.	None
Water suppr.	None
Restore magn.	Off
Freeze suppressed tissue	Off

Contrast - Dynamic

Averages	1
Averaging mode	Short term
Reconstruction	Real
Measurements	1
Multiple series	Each measurement

Resolution - Common

FoV read	256 mm
FoV phase	100.0 %
Slice thickness	6.0 mm
Base resolution	256
Phase resolution	75 %
Phase partial Fourier	Off
Trajectory	Cartesian
Interpolation	Off

Resolution - iPAT

PAT mode	None

Resolution - Filter Image

Image Filter	Off
Distortion Corr.	Off
Prescan Normalize	Off
Normalize	Off
B1 filter	Off

Resolution - Filter Rawdata

Raw filter	Off
Elliptical filter	Off

Geometry - Common

Slice group	1
Slices	1
Dist. factor	50 %
Position	L1.5 A13.4 F0.5 mm
Orientation	Sagittal
Phase enc. dir.	A >> P
FoV read	256 mm
FoV phase	100.0 %
Slice thickness	6.0 mm
TR	4500.0 ms
Multi-slice mode	Interleaved
Series	Interleaved
Concatenations	1

Geometry - AutoAlign

Slice group	1
Position	L1.5 A13.4 F0.5 mm
Orientation	Sagittal
Phase enc. dir.	A >> P
AutoAlign	Head > Basis
Initial Position	L1.5 A13.4 F0.5
L	1.5 mm
A	13.4 mm
F	0.5 mm
Initial Rotation	0.00 deg
Initial Orientation	Sagittal

Geometry - Saturation

Fat suppr.	None
Water suppr.	None
Restore magn.	Off
Special sat.	None

Geometry - Navigator

System - Miscellaneous

Positioning mode	FIX
Table position	H
Table position	0 mm
MSMA	S - C - T
Sagittal	R >> L
Coronal	A >> P
Transversal	F >> H
Coil Combine Mode	Adaptive Combine
Save uncombined	Off
Matrix Optimization	Off

System - Miscellaneous

AutoAlign	Head > Basis
Coil Select Mode	Default

System - Adjustments

B0 Shim mode	Tune up
B1 Shim mode	TrueForm
Adjust with body coil	Off
Confirm freq. adjustment	Off
Assume Dominant Fat	Off
Assume Silicone	Off
Adjustment Tolerance	Auto

System - Adjust Volume

Position	Isocenter
Orientation	Transversal
Rotation	0.00 deg
A >> P	263 mm
R >> L	350 mm
F >> H	350 mm
Reset	Off

System - pTx Volumes

B1 Shim mode	TrueForm

System - Tx/Rx

Frequency 1H	123.254412 MHz
Correction factor	1
Gain	High
Img. Scale Cor.	1.000
Reset	Off
? Ref. amplitude 1H	0.000 V

Physio - Signal1

1st Signal/Mode	None
TR	4500.0 ms
Concatenations	1

Physio - Cardiac

Magn. preparation	Slice-sel. IR
TI	3000 ms
Fat suppr.	None
Dark blood	Off
FoV read	256 mm
FoV phase	100.0 %
Phase resolution	75 %
Trajectory	Cartesian

Physio - PACE

Resp. control	Off
Concatenations	1

Inline - Common

Subtract	Off
Measurements	1
StdDev	Off
Save original images	On

Inline - MIP

MIP-Sag	Off
MIP-Cor	Off
MIP-Tra	Off
MIP-Time	Off
Save original images	On

Inline - Composing

Distortion Corr.	Off

Sequence - Part 1

Introduction	On
Dimension	2D
Compensate T2 decay	Off
Reduce Motion Sens.	Off
Contrasts	1
Flow comp.	No
Multi-slice mode	Interleaved
Free echo spacing	Off
Echo spacing	8.5 ms
Bandwidth	279 Hz/Px

Sequence - Part 2

Define	Turbo factor
Echo trains per slice	32
Phase correction	Automatic
Acoustic noise reduction	None
RF pulse type	Normal
Gradient mode	Fast
Hyperecho	Off
WARP	Off
Red. EC sensitivity	Off
Turbo factor	6

Sequence - Assistant

Mode	Off
Allowed delay	0 s
