# Supplementary material for: Intraoperative 3D quantitative magnetic resonance imaging in paediatric brain tumour surgery
Source: PLoS One. 2026 Feb 17;21(2):e0332562. doi: 10.1371/journal.pone.0332562 (PMC12912603; doi:10.1371/journal.pone.0332562)
Supplement: Attachment — Submitted filename: review.pdf [file pone.0332562.s008.pdf]

Review of “Intraoperative 3D quantitative magnetic resonance imaging in paediatric brain tumour surgery”

In this study, the authors measured R1 and R2 relaxation values using 3D QALAS MRI to evaluate it as an alternative to the gold-standard sequences that typically require longer duration. Compared with the preoperative head coil, they used flex coils for intraoperative scanning and compared the results. By placing the ROIs at various locations in the brain, they examined whether B1+ inhomogeneities also affect the R1 and R2 values. Although it has significant limitations, this study is a valuable proof-of-concept step towards using quantitative MRI parameters to assist in operative procedures.

<u>Major comments:</u>

- In relation to the gadolinium administration, it is unclear whether the authors observed any differences in R1. Since they mentioned scanning both before and after Gd contrast agent administration, there is no discussion on how this may have affected R1. Although R1 may not be significant, the statement in the patients examinations section in the results *“R2 was significantly higher in the intraoperative compared to preoperative examinations, both with and without contrast agent”* seems confusing, since Gadolinium is an R1 contrast agent and generally has less impact on R2. Which specific Gd-based contrast agent was used? The authors also did not include any information about it in the discussion.
- For the contrast agent studies, how was the B1+ difference calculated? What is the significance of -0.041 s-1 with no gadolinium contrast agent, and 0.047 s-1 with gadolinium contrast agent? The authors need to elaborate on this in the discussion.
- Although the authors discussed it as a limitation of the current study, how much time would increase if the authors had to perform active shimming? At least for the isotropic phantom samples, this comparison should be carried out to determine whether adding an active shimming step before scanning enhances the B1+ inhomogeneity and whether the R1 and R2 values under that condition are consistent with those calculated from the gold standard experiments, particularly in areas farther from the centre.
- The authors highlight a significant difference in R2 pre-operatively and intraoperatively as the main finding. However, apart from the absolute values, it is essential to specify the percentage difference observed and clarify what constitutes a 'significant difference,' given that the R2 values are at least one order of magnitude higher than R1. The authors should also elaborate on this aspect. While I agree the manuscript would benefit from the authors’ reflections on why a significant change in R2 was observed in vivo, the current emphasis on

anaesthesia, mannitol, and fibre tract orientation seems too hypothetical, especially in the absence of any tests done for these factors.

- In the section on the treatment of cerebral oedema, the authors mention "*there are no previous reports on the relaxation effects of mannitol (or other osmotic agents) in vivo*." I think they need to clarify that no experimentation in this study specifically tests for these effects. From a discussion perspective, I believe the section on anaesthesia and mannitol can be combined, since the detailed discussion on them appears to be the authors proposing explanations based on the experiments shown in this study, which is not the case.
- Based on the experiments conducted in this study, how do the authors envisage the calculation of R1 and R2 using 3D QALAS being applied to determining tumour volume for resection or estimating myelin concentration? This explanation should be included in the conclusion, as the current statement is too vague for a quantitative study.
- Figure 2 caption: "*The 3D-QALAS sequence consists of five dynamics*." Dynamics of what? The sentence is unclear. Also, it is not mentioned what the blue-to-orange scale represents. Assuming it is signal intensity, can the range be made the same for pre-operative and intraoperative measurements for better comparison?
- Figure 4a. Regarding the R1 and R2 correlation, are any specific clustering patterns observed, such as based on distance from the magnet centre for particular brain regions, according to specific ROIs? Currently, it is unclear whether the ROIs shown on the left are similar regions in different OP conditions.
- Figure 5. From the figure, it is unclear whether the average bias is the same for all four phantoms with different concentrations, or if the average bias is based on a specific concentration or patient data, with the phantom values overlaid to show the pattern.
- Any comments and citing the references regarding the expected B1+ in relation to flip angle would benefit the manuscript, since this factor has been used in the experiments and results in Figure 6.
- Labelling the A70 to P70 positions on an MRI scan and including them in a supplementary figure will be helpful, since the current representative figure 7 does not show the positions of all 15 ROIs.

<u>Minor comments:</u>

- Clarify what differences were observed between the head coil and flex coil. Were those restricted to just SNR differences?
- The authors mentioned that the R1 and R2 values can assist in tissue classification and estimating myelin concentration both in the introduction and discussion. Based on the experiments conducted, did the authors attempt to calculate or estimate these values for the 10 patients? If yes, what were the

results? If no, what are the limitations? If this is outside the scope of the study, they should simply mention it in the discussion.

- For the in-house MATLAB code, a reference or citation to previous reports or a GitHub link should be provided.
- While the phantoms are isotropic and the authors pointed out this limitation in the discussion, they should also suggest better alternatives for future experiments in this study. Not only should the anisotropic nature of the brain be considered, but also the presence of R2-impacting ions like iron and zinc.
- The discussion on anaesthesia affecting the increase of T2 is interesting; however, there are no experiments in this study conducted to test or support this claim. Therefore, a dedicated discussion on this point seems somewhat of a stretch, especially since the anisotropy of brain tissue structure and mineral content with ions affecting R1 and R2 are not discussed at all. Similarly, unless the authors tested for fibre tract orientation or analysed their data to be relevant, the discussion does not seem applicable to the findings.
- What do the authors interpret as the reason for the difference in their findings being due to the prone position in the intraoperative setting?
- The detail in the data analysis part "*The ROIs were placed by an experienced (15+ years) neuroradiologist (IB) on*" would be better suited to the acknowledgements or contribution section.

The study is a promising proof-of-concept, but the manuscript's writing is disjointed. Refining the purpose, experiments, and discussion will enhance clarity. Basic formatting improvements are recommended, such as adding full stops where they are missing in the introduction and methods sections.
