# Supplementary material for: Intraoperative 3D quantitative magnetic resonance imaging in paediatric brain tumour surgery
Source: PLoS One. 2026 Feb 17;21(2):e0332562. doi: 10.1371/journal.pone.0332562 (PMC12912603; doi:10.1371/journal.pone.0332562)
Supplement: Attachment — Submitted filename: plosone.pdf [file pone.0332562.s010.pdf]

The authors have addressed my concerns and taken my suggestions into account to my satisfaction. I appreciate the potential of this study in intraoperative diagnostic imaging in paediatric brain surgery.

There are minor language suggestions that the authors can consider enhancing the clarity of the manuscript, which they may address at their discretion.

1. Abstract : Last line under the conclusion heading, change “R2 tissue values” to “R2 values in tissue”.
2. Introduction: The sentence “Concerning paediatric brain tumours there is little or no knowledge concerning the use of qMRI and to our knowledge none in the perioperative setting during paediatric brain tumour surgery”, could be rephrased to “*Concerning pediatric brain tumors, there is little to no knowledge about the use of qMRI, and, to our knowledge, none in the perioperative setting during pediatric brain tumor surgery*” for clarity.

“And also that R1 and R2 measurements were affected by different receive coils. ” should be rephrased to “*Additionally, it was examined whether the R1 and R2 measurements were affected by different receiver coils*”.

3. Results: The previous section title “*Patient Examination*” was better than “patient”. Last line in this section “N.B.” can be changed to “Please note”. The previous section title “*Relaxation measurement in phantoms*” was better than “phantoms”.
4. Discussion: Under the factors affecting R1 and R2 section, the sentence “In the following we therefore attempt to explore which factors, whether chemical of physical, that potentially would explain the observations.” A word seems to be missing after ‘following’. The sentence could be replaced with “*In the following paragraphs, we explain the potential chemical and physical phenomena behind these observations*.”

Since there is only one sub-heading after this paragraph, either the title “chemical effectors and structure of brain tissue” could be removed, or an appropriate heading like “Anaesthesia” could be given to the context in the following paragraphs.
