# Supplementary material for: Intraoperative 3D quantitative magnetic resonance imaging in paediatric brain tumour surgery
Source: PLoS One. 2026 Feb 17;21(2):e0332562. doi: 10.1371/journal.pone.0332562 (PMC12912603; doi:10.1371/journal.pone.0332562)
Supplement: Attachment — Submitted filename: (B) Response to reviewers IntraOP_QALAS_PlosOne.docx [file pone.0332562.s009.docx]

PONE-D-25-46877] Review of “Intraoperative 3D quantitative magnetic resonance imaging in paediatric brain tumour surgery”

***REVIEWER #1***

***REVIEWER #1-1:*** *In "Intraoperative 3D quantitative magnetic resonance imaging in paediatric brain tumour surgery" the authors investigate in both phantoms and surgical patients the change in R1 and R2 between preoperative and interoperative qMRI. The authors find that in phantoms and in vivo, R1 is consistent between pre- and interoperative scanning, but R2 increases in vivo. Because phantom R2 is the same between both scanning situations, the authors attribute change in R2 to physiologic changes during surgery.*

*Although the work is very good, I have to recommend major revision, as Table 1 was not included in the manuscript, and thus could not be reviewed.*

**RESPONSE**: Our apologies. Table 1 has now been added to the manuscript.

***REVIEWER #1-2:*** *Generally, figures should be numbered in order of their appearance. The present order of first mention of figures appears to be 1, 2, 3, 7, 6, 4, 5,*

**RESPONSE**: Our apologies. We have improved the flow of the presentation in the revision, including the sequence of the figures, which hopefully will facilitate reading. The main challenge has been to focus on either phantom or patient measurements, whenever such is most relevant in the flow of the manuscript.

***REVIEWER #1-3:*** *P4, Magnetic Resonance, Data acquisition section, paragraph 1: Figure 1 does not contain what it is stated to contain in this paragraph. Perhaps this is meant to refer to what is presently Figure 3?*

**RESPONSE**: Our apologies. The text referring to Fig 1 was adjusted and also improved, and similarly with the corresponding figure legend. Figure 1 describes the overall structure of the work, ranging from coils and ROIs to the analysis.

***REVIEWER #1-4:*** *P5, Patient MR Measurements, paragraph 4: Table 1 cannot be found.*

**RESPONSE**: Table 1 has been attached. It contains age, sex, duration, position, medication preoperative, medication intraoperative, and diagnosis for all included patients. The table also provides a basic view of the prospective nature of the study.

***REVIEWER #2***

***REVIEWER #2:*** *In this study, the authors measured R1 and R2 relaxation values using 3D QALAS MRI to evaluate it as an alternative to the gold-standard sequences that typically require longer duration. Compared with the preoperative head coil, they used flex coils for intraoperative scanning and compared the results. By placing the ROIs at various locations in the brain, they examined whether B1+ inhomogeneities also affect the R1 and R2 values. Although it has significant limitations, this study is a valuable proof-of- concept step towards using quantitative MRI parameters to assist in operative procedures.*

**RESPONSE**: We appreciate that you sense the significant potential and novelty of this work. Something that has encouraged us to express it more clearly.

*Major comments:*

***REVIEWER #2_1:*** *In relation to the gadolinium administration, it is unclear whether the authors observed any differences in R1. Since they mentioned scanning both before and after Gd contrast agent administration, there is no discussion on how this may have affected R1.*

*Although R1 may not be significant, the statement in the patients examinations section in the results “R2 was significantly higher in the intraoperative compared to preoperative examinations, both with and without contrast agent” seems confusing, since Gadolinium is an R1 contrast agent and generally has less impact on R2.*

*Which specific Gd-based contrast agent was used? The authors also did not include any information about it in the discussion.*

**RESPONSE**:

Yes, it is correct that R1 is affected by Gd^3+^ based CA stronger than R2. However, the aim of this work was to investigate how the measurement of R1 and R2 are affected *by the intraoperative setting*, compared to the preoperative setting. For that reason, we paired ROIs from the pre- and intra examination without Gd^3+^ CA and with Gd^3+^ CA, separately. Thus, we did not investigate how Gd^3+^ CA affected R1 or R2. Based on such analysis we could report how Gd^3+^ CA affected R1 or R2, which we agree is an interesting question *per se*, although outside the scope of this work. It is nevertheless worth observing that the differences of R1, R2 and B1, respectively, were similar for both native and Gd^3+^ CA determined parameters.

Finally, the specific contrast agent used was *Dotarem* (gadoterate meglumine), which is described in the Methods section.

***REVIEWER #2_2:*** *For the contrast agent studies, how was the B1+ difference calculated? What is the significance of -0.041 s-1 with no gadolinium contrast agent, and 0.047 s-1 with gadolinium contrast agent? The authors need to elaborate on this in the* *discussion.*

**RESPONSE**: Thank you for spotting this error. Both B1+ differences have the same sign, that is –0.041 s-1 (w/o Gd CA), and –0.047 s-1 (w Gd CA). B_1_^+^ was calculated from measured B_1_^+^ maps. The maps were then transformed into 3D QALAS space, and the appropriate ROIs were used to determine the differences.

***REVIEWER #2_3:*** *Although the authors discussed it as a limitation of the current study, how much time would increase if the authors had to perform active shimming? At least for the isotropic phantom samples, this comparison should be carried out to determine whether adding an active shimming step before scanning enhances the B1+ inhomogeneity and whether the R1 and R2 values under that condition are consistent with those calculated from the gold standard experiments,* *particularly in areas farther from the centre.*

**RESPONSE**: This is an interesting aspect, with respect B_1_^+^ homogeneity. It is certainly possible that 'Active shimming' could improve the homogeneity, at least far from the isocentre. However, so called '*Patient specific shimming*' (using Siemens terminology) is not included in the clinical protocols, on which this work is based, and were therefore not included here. In the future, we could consider including it as it might improve the B_1_^+^ homogeneity at far distances (off centre), but not necessarily closer to the iso-centre.

***REVIEWER #2_4:*** *The authors highlight a significant difference in R2 pre-operatively and* *intraoperatively as the main finding. However, apart from the absolute values, it is essential to specify the percentage difference observed and clarify what* *constitutes a 'significant difference,' given that the R2 values are at least one order of magnitude higher than R1. The authors should also elaborate on this* *aspect.*

*While I agree the manuscript would benefit from the authors’ reflections on why a significant change in R2 was observed in vivo, the current emphasis on anaesthesia, mannitol, and fibre tract orientation seems too hypothetical, especially in the absence of any tests done for these factors.*

**RESPONSE**: We agree that R2 is an order of magnitude larger than R1. However, the *change* in R2 was almost two orders of magnitude larger than the change in R1. The change in R2 was more than 10% while the (non-significant) change in R1 was ten times less (around 1%), thus R2 seems to be more affected, even in relative terms. We have clarified this in the result section of the manuscript.

Based on the finding of difference of R2 between pre- and intra-operative settings we have attempted to evaluate possible influences of different physical and chemical factors that might be active. However, at this time we do not have access to data that would clarify actual causalities.

***REVIEWER #2_5:*** *In the section on the treatment of cerebral oedema, the authors mention “there are no previous reports on the relaxation effects of mannitol (or other osmotic agents) in vivo." I think they need to clarify that no experimentation in this study specifically tests these effects. From a discussion perspective, I believe the section on anaesthesia and mannitol can be combined, since the detailed discussion on them appears to be the authors proposing explanations based on the experiments shown in this study, which is not the case.*

**RESPONSE**: This has now been described more clearly in the Discussion, *i.e.*, that such experiments have not yet been performed, neither in our previous, nor in this work.

***REVIEWER #2_6:*** *Based on the experiments conducted in this study, how do the authors envisage the calculation of R1 and R2 using 3D QALAS being applied to determining tumour volume for resection or estimating myelin concentration? This explanation should be included in the conclusion, as the current statement is too vague for a quantitative study.*

**RESPONSE**: In our previous work (Blystad et al, *Quantitative MRI for analysis of peritumoral edema in malignant gliomas.* PLoS One. 2017;12(5):e0177135) we have shown that qMRI could be used for detecting tumour infiltration in the oedema. A long-term goal is to investigate if these results can also be applied in an intraoperative qMRI. However, there are several factors such as brain shift, B_1_^+^ inhomogeneity, the use of flex coils, etc. that could influence the measurements of R1 and R2 in the intraoperative setting. For that reason, we have in this work focused on the normal appearing matter, in which we did not expect any differences in relaxation values.

***REVIEWER #2_7:*** *Figure 2 caption: “The 3D-QALAS sequence consists of five dynamics.” Dynamics of what? The sentence is unclear.*

*Also, it is not mentioned what the blue-to- orange scale represents. Assuming it is signal intensity, can the range be made the same for pre-operative and intraoperative measurements for better comparison?*

**RESPONSE**: The figure legend for Fig 3(A) was rephrased. ‘Dynamics’ is the conventional term often used with the 3D QALAS pulse sequence. The 3D-QALAS sequence consists of five ‘dynamics’ (see top panel where the five points for data sampling are marked with green squares). Each dynamic consists of a preparation phase and an acquisition phase. In the first dynamic, a T2 preparation pulse is used to make the first volume T2 weighted. The second dynamic starts with an 180 degree-inversion pulse to encode R1 relaxation. In all dynamics, the acquisitions are read with a 4-degree flash readout.

The blue to yellow scale represents an imaging scale for signal intensities spanning from minimum to maximum intensity; a dynamic (and not a global fixed) scale was thus used in the two panels representing pre-op and intra-op, respectively.

***REVIEWER #2_8:*** *Figure 4a. (Ed: now Fig 5a) Regarding the R1 and R2 correlation, are any specific clustering patterns observed, such as based on distance from the magnet centre for particular brain regions, according to specific ROIs? Currently, it is unclear whether the ROIs shown on the left are similar regions in different OP conditions.*

**RESPONSE**: This is an interesting suggestion. It was initially considered but has not been done. The reason was that the exact orientation of the patient’s heads was not known with respect to the scanner coordinate system. Our attempt to extract the positioning unfortunately failed because of the unclear registration of head geometry with respect to the scanner coordinate system, as it (possibly) is stored in the DICOM parameters.

***REVIEWER #2_9:*** *Figure 5 (Ed. Now, Fig 6). From the figure, it is unclear whether the average bias is the same for all four phantoms with different concentrations, or if the average bias is based on a specific concentration or patient data, with the phantom values overlaid to show the pattern.*

**RESPONSE**: Our apologies, we understand the confusion. For the four different phantoms we used the same description (i.e., the average bias), which may not be the most appropriate as only the phantoms with lowest concentrations are those that are physiologically most relevant. We have now added an additional line in the diagram, at the position for the maximal R (or R2) for the *in vivo* situation.

***REVIEWER #2_10:*** *Any comments and citing the references regarding the expected B1+ in relation to flip angle would benefit the manuscript, since this factor has been used in the experiments and results in Figure 6 (Ed: Now Fig S1 in the Supplement).*

**RESPONSE**: We agree that this can be described more clearly. The B_1_^+^ scale reported by the scanner is normalized to 1.0 for the desired FA. Values less than 1.0 means a smaller actual FA, than the nominal, and correspondingly larger values represent a larger actual FA. This is also discussed in more detail in Ref 19, in particular in Eq. 2 in the reference, in which the relation between the 'B_1_^+^-factor' and FA is described. The measured B_1_^+^ factor thus corresponds to how much the nominal FA deviates from the actual FA. Text was rephrased to explain this.

*Minor comments:*

***REVIEWER #2_11:*** *Labelling the A70 to P70 positions on an MRI scan and including them in a supplementary figure will be helpful, since the current representative figure 7 (Ed. Now Fig S1) does not show the positions of all 15 ROIs.*

**RESPONSE**: An additional figure has been added to the Supplement (see Fig S3). This figure shows 15 ROI positions.

***REVIEWER #2_12:*** *Clarify what differences were observed between the head coil and flex coil. Were those restricted to just SNR differences?*

**RESPONSE**: Correct that there is a notable SNR difference between these two coil settings. However, R1 and R2 differences were not affected SNR as far as we could observe, for example as is shown in Figs S1 and S2. Columns one and two in the figures are very similar, showing that there were no obvious differences.

***REVIEWER #2_13:*** *The authors mentioned that the R1 and R2 values can assist in tissue classification and estimating myelin concentration both in the introduction and discussion. Based on the experiments conducted, did the authors attempt to calculate or estimate these values for the 10 patients? If yes, what were the results? If no, what are the limitations? If this is outside the scope of the study, they should simply mention it in the discussion.*

**RESPONSE**: We agree that in this work determining the myelin concentrations were outside the scope of this work. This was added to the Discussion, for more clarity.

***REVIEWER #2_14:*** *For the in-house MATLAB code, a reference or citation to previous reports or a GitHub link should be provided.*

**RESPONSE**: We have added the MatLab code (*Code.zip*) for the fitting procedures, see Supplement.

***REVIEWER #2_15:*** *While the phantoms are isotropic and the authors pointed out this limitation in the discussion, they should also suggest better alternatives for future experiments in this study. Not only should the anisotropic nature of the brain be considered, but also the presence of R2-impacting ions like iron and zinc.*

**RESPONSE**: Thank you for the suggestion. We have rephrased the Discussion slightly to encompass this suggestion, although at this time we have no working or practical solutions for a suitable anisotropic phantom.

***REVIEWER #2_16:*** *The discussion on anaesthesia affecting the increase of T2 is interesting; however, there are no experiments in this study conducted to test or support this claim. Therefore, a dedicated discussion on this point seems somewhat of a stretch, especially since the anisotropy of brain tissue structure and mineral content with ions affecting R1 and R2 are not discussed at all.*

*Similarly, unless the authors tested for fibre tract orientation or analysed their data to be relevant, the discussion does not seem applicable to the findings.*

**RESPONSE**: Thank you for these suggestions, and we are aware of the possible importance of these anisotropy and ionic agent distribution effects. We have put less emphasis on these effects in the Discussion. To test fiber tract orientations in patient subjects is not within ethical reach at this time, as it would affect the surgical treatment.

***REVIEWER #2_17:*** *What do the authors interpret as the reason for the difference in their findings being due to the prone position in the intraoperative setting?*

**RESPONSE**: We have not analysed the data and the effects of the positioning of each individual patient. One main reason being that each patient is positioned in the most optimal position for the surgical procedure for the individual patient. However, we have put quite some efforts in how to devise suitable analysis methods to be able to achieve such analysis. Unfortunately, at this time we have no clear path on how to extract and analyse the available geometry data in such a manner.

***REVIEWER #2_18:*** *The detail in the data analysis part “The ROIs were placed by an experienced (15+ years) neuroradiologist (IB) on” would be better suited to the acknowledgements or contribution section.*

**RESPONSE**: In our experience, this information is often requested by reviewers, at least in our experience. Which is the reason why it was mentioned in the current context.

***REVIEWER #2_19:*** *The study is a promising proof-of-concept, but the manuscript's writing is disjointed. Refining the purpose, experiments, and discussion will enhance clarity. Basic formatting improvements are recommended, such as adding full stops where they are missing in the introduction and methods sections.*

**RESPONSE**: The manuscript has been language edited by a professional British speaking editor. We have now also re-checked the entire text with these comments in mind.

Finally, we would like to thank both reviewers sincerely for many valuable comments.
