# Supplementary material for: Intraoperative 3D quantitative magnetic resonance imaging in paediatric brain tumour surgery
Source: PLoS One. 2026 Feb 17;21(2):e0332562. doi: 10.1371/journal.pone.0332562 (PMC12912603; doi:10.1371/journal.pone.0332562)
Supplement: Attachment — Submitted filename: Response to reviewer 2 IntraOP_QALAS_PlosOne.docx [file pone.0332562.s011.docx]

PONE-D-25-46877] Review of “Intraoperative 3D quantitative magnetic resonance imaging in paediatric brain tumour surgery”

***REVIEWER #2***

All issues were addressed.

We would like to thank the reviewer sincerely for valuable comments.
